# Inhibition of JNK signaling in the Asian malaria vector *Anopheles stephensi* extends mosquito longevity and improves resistance to *Plasmodium falciparum* infection

**DOI:** 10.1371/journal.ppat.1007418

**Published:** 2018-11-29

**Authors:** Lattha Souvannaseng, Lewis Vibul Hun, Heather Baker, John M. Klyver, Bo Wang, Nazzy Pakpour, Jordan M. Bridgewater, Eleonora Napoli, Cecilia Giulivi, Michael A. Riehle, Shirley Luckhart

**Affiliations:** 1 Department of Medical Microbiology and Immunology, University of California Davis, Davis, CA, United States of America; 2 Department of Pathobiology, St. George's University, School of Veterinary Medicine, True Blue, St. George, Grenada, West Indies; 3 Department of Entomology, University of Arizona, Tucson, AZ, United States of America; 4 Department of Molecular Biosciences, University of California, Davis, Davis, CA; 5 M.I.N.D. Institute, Sacramento, CA, United States of America; 6 Department of Entomology, Plant Pathology and Nematology and Department of Biological Sciences, University of Idaho, Moscow, ID, United States of America; Stanford University, UNITED STATES

## Abstract

Malaria is a global health concern caused by infection with *Plasmodium* parasites. With rising insecticide and drug resistance, there is a critical need to develop novel control strategies, including strategies to block parasite sporogony in key mosquito vector species. MAPK signaling pathways regulated by extracellular signal-regulated kinases (ERKs) and the stress-activated protein kinases (SAPKs) *c*-Jun N-terminal kinases (JNKs) and p38 MAPKs are highly conserved across eukaryotes, including mosquito vectors of the human malaria parasite *Plasmodium falciparum*. Some of these pathways in mosquitoes have been investigated in detail, but the mechanisms of integration of parasite development and mosquito fitness by JNK signaling have not been elucidated. To this end, we engineered midgut-specific overexpression of MAPK phosphatase 4 (MKP4), which targets the SAPKs, and used two potent and specific JNK small molecule inhibitors (SMIs) to assess the effects of JNK signaling manipulations on *Anopheles stephensi* fecundity, lifespan, intermediary metabolism, and *P*. *falciparum* development. MKP4 overexpression and SMI treatment reduced the proportion of *P*. *falciparum-*infected mosquitoes and decreased oocyst loads relative to controls. SMI-treated mosquitoes exhibited no difference in lifespan compared to controls, whereas genetically manipulated mosquitoes exhibited extended longevity. Metabolomics analyses of SMI-treated mosquitoes revealed insights into putative resistance mechanisms and the physiology behind lifespan extension, suggesting for the first time that *P*. *falciparum*-induced JNK signaling reduces mosquito longevity and increases susceptibility to infection, in contrast to previously published reports, likely via a critical interplay between the invertebrate host and parasite for nutrients that play essential roles during sporogonic development.

## Author summary

Malaria is a global health concern caused by infection with *Plasmodium* parasites. With rising insecticide and drug resistance, there is a critical need to develop novel control strategies. One strategy is to develop a *Plasmodium*-resistant mosquito through the manipulation of key signaling pathways and processes in the mosquito midgut, a critical tissue for parasite development. MAPK signaling pathways are highly conserved among eukaryotes and regulate development of the human malaria parasite *Plasmodium falciparum* in the mosquito vector. Here, we investigated how manipulation of *Anopheles stephensi* JNK signaling affects development of *P*. *falciparum* and key mosquito life history traits. We used multiple, complementary approaches to demonstrate that malaria parasite infection activates mosquito JNK signaling for its own benefit at a cost to host lifespan. Notably, these combined effects derive from networked signaling with other transduction pathways and alterations to intermediary metabolism in the mosquito host.

## Introduction

The etiologic agents of malaria are protozoan parasites in the genus *Plasmodium* and are responsible for 216 million new cases and 445,000 deaths worldwide in 2016 [1]. Artemisinin-based combination therapies (ACTs) have been adopted as first-line treatments of uncomplicated and severe *Plasmodium falciparum* malaria in many countries with concomitant reductions in the global malaria burden [2]. Unfortunately, artemisinin-resistant malaria parasites have been detected in five countries in Southeast Asia [3] and spread of these strains to Africa or the Indian subcontinent could be disastrous. *Anopheles stephensi*, one of the major vectors of malaria in the Indian subcontinent and Middle East, is well adapted to urban areas and feeds aggressively on humans. The appearance of *A*. *stephensi* in Djibouti, Horn of Africa, has been linked to a recent resurgence of severe *Plasmodium falciparum* malaria [4]. More recently, *A*. *stephensi* has been detected in Sri Lanka, where it has never been reported, raising concerns regarding vulnerability of this country to reintroduction of malaria [5]. Therefore, continued efforts in the development of novel strategies and tools to curb malaria transmission, including those focused on the mosquito host, are still required.

During sporogony, parasites encounter an array of impediments within the mosquito that can limit infection. Innate immune pathways include the Toll signaling cascade [6], the Janus kinase/signal transducers and activators of transcription (JAK/STAT) [7], and the immune deficiency (IMD) pathway [8]. Parasite activation of these signaling pathways results in the synthesis of antimicrobial peptides, reactive nitrogen and oxygen species (RNOS), and other immune factors (e.g., TEP1, APL1, LRIM1, LRRD7) that are anti-parasitic [9, 10]. Signaling proteins and pathways that finely tune mosquito defense against parasite infection include the mitogen activated protein kinases (MAPKs) [11, 12], insulin/insulin-like growth factor signaling (IIS) [13–16], and the transforming growth factor (TGF)-β signaling pathway [17]. These pathways regulate mosquito NO synthase (NOS) and production of RNOS [16, 18, 19] as well as intermediary metabolism and epithelial barrier function in the mosquito midgut to control parasite infection [12, 16, 20–22].

The stress-associated protein kinases (SAPKs), including the *c*-Jun N-terminal kinases (JNKs) and p38 MAPKs, along with the extracellular signal-regulated kinases (ERKs) are members of the mitogen activated protein kinase (MAPK) superfamily. Mammalian JNK1, JNK2, and JNK3 are encoded by three different genes, all of which are associated with several alternatively spliced products (e.g., JNK1A). The single JNK ortholog in *Drosophila melanogaster*, Basket, triggers apoptosis in embryonic epithelia during normal development as well as in response to γ-irradiation [23]. Unlike *D*. *melanogaster*, the *Anopheles gambiae* genome encodes two JNK orthologs AGAP009461 and AGAP009460 that are homologous to mammalian JNK1 and JNK3, respectively [24]. The *A*. *stephensi* genome encodes JNK1 (ASTE007551) and JNK3 (ASTE007552) that are in 1:1 orthology with *A*. *gambiae* JNK1 and JNK3.

JNK signaling has been implicated in mosquito defense against malaria parasites. In *A*. *stephensi*, human insulin-like growth factor 1 (IGF1) induces *NOS* expression to mediate inhibition of *P*. *falciparum* development via enhanced JNK activation in the midgut epithelium [20]. In *A*. *gambiae*, JNK activation has been linked to the regulation of heme peroxidase 2 and NAPDH oxidase 5, both of which function with NOS to opsonize parasites, leading to TEP1-mediated complement-like elimination in the mosquito host [25]. Other work has suggested that the *P*. *falciparum* protein *Pfs*47, expressed in the ookinete stage, disrupts *A*. *gambiae* JNK signaling to facilitate parasite evasion of the immune response and enhance parasite survival in the mosquito [26]. In related work, RNAi-dependent gene silencing of the *A*. *gambiae* ortholog of *puckered*, a MAPK phosphatase (MKP) that negatively regulates Basket in *Drosophila*, enhanced resistance against *Plasmodium berghei* infection in *A*. *gambiae* [27].

Gene products that impact *Plasmodium* development make attractive targets for genetic modification to enhance immune resistance against malaria parasite infection. Indeed, overexpression of the IIS kinase Akt in the *A*. *stephensi* midgut epithelium resulted in complete refractoriness to *P*. *falciparum* infection in this mosquito host [13]. However, genetic manipulation can negatively impact key fitness traits such as lifespan and reproductive output. While this may be effective at reducing mosquito “infective lifespan” and preventing parasite development, modifications that decrease lifetime fecundity also limit the fitness of modified mosquitoes relative to unmodified natural populations. Perhaps more importantly, however, high fitness costs can reduce the efficiency of genetic drive mechanisms to fix transgenes into mosquito populations, preventing successful replacement of susceptible mosquito populations with those that are resistant to parasite infection.

Evidence from *D*. *melanogaster* has demonstrated that moderate inhibition of the JNK signaling cascade, which regulates lifespan and immunity through midgut homeostasis, can mitigate the fitness costs of enhanced immunity. JNK activity induces stem cell proliferation in the fly midgut, but chronic signaling contributes to loss of tissue homeostasis in aged flies, particularly when exposed to high stress [28]. Reducing JNK activity prevented the age-associated changes in midgut physiology and improved resistance to stress. These observations indicated that age-related gut epithelium homeostasis, lifespan, and immune function are regulated, at least in part, by JNK signaling, physiology that is likely evident in related Diptera, including mosquito vector species in the genus *Anopheles*. To test this hypothesis, we utilized genetic manipulation of midgut SAPK signaling and potent JNK-specific small molecule inhibitors (SMIs) to test the hypothesis that parasite resistance and mosquito fitness are coordinately regulated by this pathway in *A*. *stephensi*. Both strategies inhibited JNK activity in the midgut epithelium, reduced the proportion of *P*. *falciparum-*infected mosquitoes and decreased oocyst loads relative to controls. SMI-treated mosquitoes exhibited no difference in lifespan compared to controls with modest effects on fecundity, whereas genetically manipulated mosquitoes exhibited increased longevity and reduced fecundity with increasing JNK signaling inhibition. Further, manipulation of JNK signaling with SMIs suggested that *P*. *falciparum* increases the accessibility of previously identified essential nutrients from its mosquito host via activation of this pathway. These results provide new insights into JNK regulation of mosquito physiology and vector competence and elucidate new mechanisms whereby mosquito life history traits are intimately connected with resistance to parasite infection.

## Results

### Patterns of JNK activation with age and during *P*. *falciparum* infection in *A*. *stephensi*

**Fig 1:**
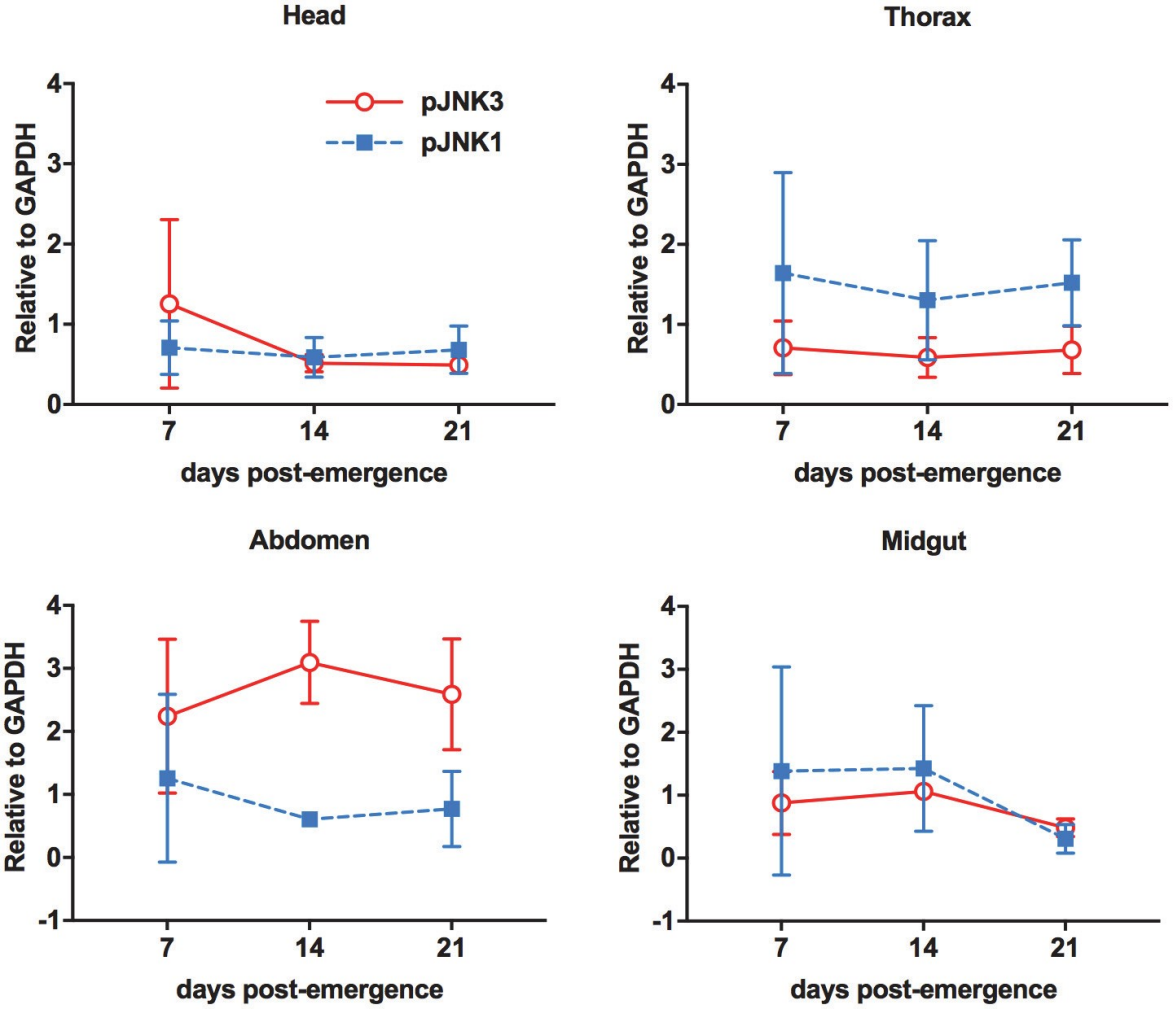
Relative levels of *A*. *stephensi* JNK1 and JNK3 phosphorylation varied among mosquito tissues but did not change with age. Heads, thoraces, abdomens, and midguts from 15 female *A*. *stephensi* maintained on 10% sucrose were processed for western blotting at days 7, 14, and 21 post-emergence. Levels of phosphorylated JNK1 and JNK3 were normalized to GAPDH levels in the same samples. This experiment was replicated with four separate cohorts of mosquitoes. Expression levels relative to GAPDH between timepoints were analyzed by Student’s t-test.

JNK signaling increases with age in the midgut and is required for stress tolerance in midgut epithelial cells in *D*. *melanogaster* [28]. To determine if the same pattern was evident in mosquitoes, phosphorylated JNK (pJNK) levels were assessed in the head, thorax, abdomen, and midgut tissues of female *A*. *stephensi* at 7, 14, and 21 days-post-emergence. Relative pJNK1 and pJNK3 levels varied among mosquito tissues, but contrary to observations in *D*. *melanogaster*, changed little with age (**Fig 1**).

**Fig 2:**
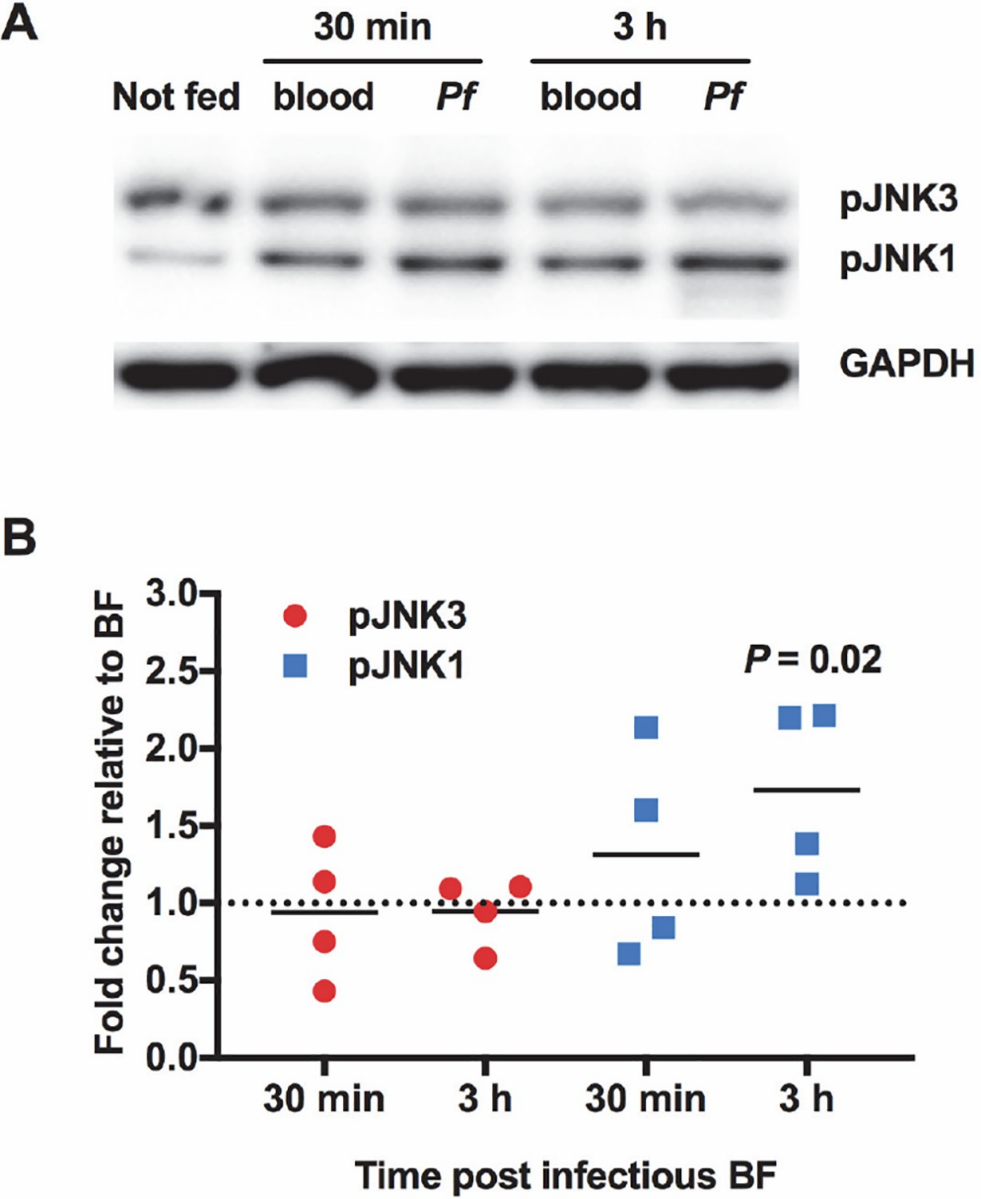
JNK1 was activated in the *A*. *stephensi* midgut in response to *P*. *falciparum* infection. **(A)** Representative western blot. Female *A*. *stephensi* mosquitoes (3–5 day old) were provided a meal of *P*. *falciparum*-infected red blood cells (*Pf*) or a meal of uninfected red blood cells (blood). Midguts were dissected at 30 min and 3 h post-feed and processed for western blotting. Levels of GAPDH in the midgut were used to assess loading and for normalization. **(B)** Levels of phosphorylated JNK1 and JNK3 in midguts of infected *A*. *stephensi* were normalized to levels in mosquitoes fed uninfected blood (set at 1.0, dotted line). This experiment was replicated with four separate cohorts of mosquitoes. Fold changes relative to mosquitoes fed uninfected blood at each timepoint were analyzed by Student’s t-test.

To determine whether JNK was activated in response to malaria parasite infection, female *A*. *stephensi* were fed a *P*. *falciparum-*infected blood meal and midgut JNK phosphorylation levels were quantified at 30 min and 3 h post-blood feeding. The results revealed a trend towards increased JNK1 phosphorylation in mosquitoes fed *P*. *falciparum*-infected red blood cells (RBCs) at 30 min post-feeding compared to mosquitoes fed RBCs alone, and a significant increase in JNK1 phosphorylation at 3 h post-feeding (**Fig 2**). JNK3 phosphorylation levels remained at baseline for both time points (**Fig 2**).

### Identification and phylogenetic analysis of *A*. *gambiae* and *A*. *stephensi* MKPs

**Fig 3:**
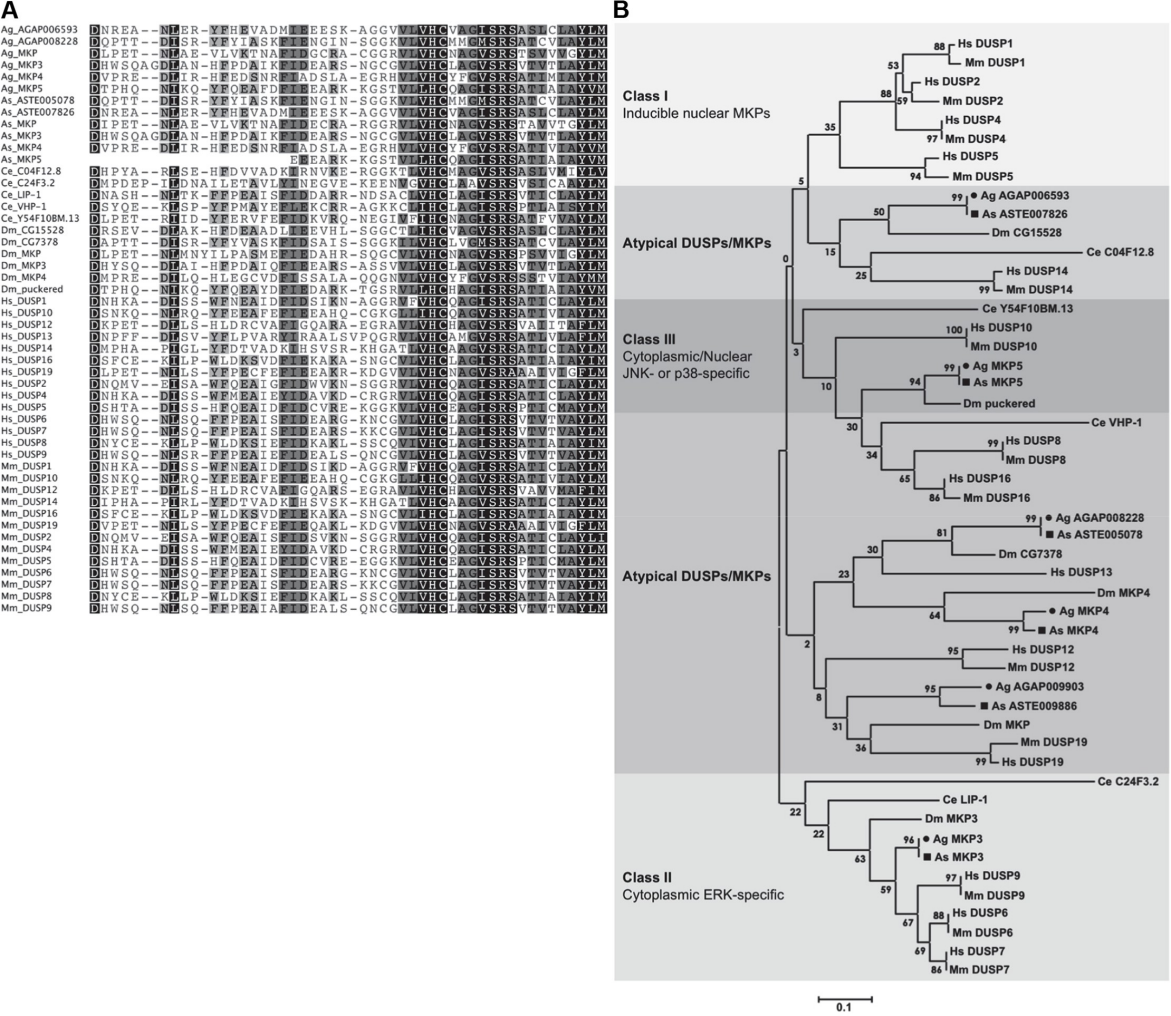
Mosquito MKPs are homologous to fruit fly, human, and mouse MKPs. **(A)** *A*. *gambiae* (Ag) and *A*. *stephensi* (As) MKPs possess the highly conserved extended active site sequences and residues required for MKP catalysis, **D**X_26_VLVH**C**X(A/M)G(V/I)S**R**SX_5_AYLM, suggesting that the encoded proteins are enzymatically active. **(B)** Phylogenetic tree based on MKP catalytic domain sequences. Highly homologous mosquito MKP proteins are grouped with class II, class III, and atypical DUSPs. Class I MKPs are not encoded in the *A*. *gambiae*, *A*. *stephensi*, *D*. *melanogaster* or *C*. *elegans* genomes. Circles and squares denote *A*. *gambiae* and *A*. *stephensi* MKP proteins, respectively.

For MAPKs to be active, both the threonine and tyrosine residues in the conserved signature sequence threonine-x-tyrosine (TxY) within the activation motif must be phosphorylated, typically by an upstream MAPK kinase [11]. Thus, removal of a single phosphate from either motif is sufficient for MAPK inactivation. The MKPs, which are dual-specificity phosphatases (DUSPs) and members of the protein tyrosine phosphatase (PTP) superfamily, render MAPKs inactive by catalyzing the removal of both phosphates from the activation motif [29–31]. All MKPs contain a highly conserved *C*-terminal catalytic domain with the extended consensus signature motif **D**X_26_VLVH**C**X(A/M)G(V/I)S**R**SX_5_AYL with the residues in bold essential for catalysis [30, 32]. Six MKPs have been identified in the *D*. *melanogaster* genome, all of which contain the extended conserved consensus motif [33–36]. The *Anopheles* MKPs also possess the highly conserved extended active site sequences and residues required for MKP catalysis, suggesting that the encoded proteins are enzymatically active (**Fig 3A**). MKPs are grouped as class I, II, III or atypical MKPs based on subcellular localization and substrate specificities (summarized in **Table 1**) [37]. Phylogenetic analysis revealed that *A*. *gambiae* AGAP012237 and *A*. *stephensi* ASTE011534 grouped with class II MKPs, and that AGAP004353 and ASTE002907 grouped with class III MKPs. AGAP002108 and ASTE004228 aligned closely with DUSP12, and AGAP009903 and ASTE009886 aligned closely with DUSP19, both of which are considered atypical DUSPs (**Fig 3B**). The mosquito MKP genes have 1-to-1 orthology with *D*. *melanogaster* genes and, therefore, are named similarly (**Table 1**).

**Table 1:** List of vertebrate, fly, and mosquito MKP genes.

Vertebrate*		*D*. *melanogaster*	*A*. *gambiae*		*A*. *stephensi*		
Gene	Synonym	Gene	Gene	Protein	Gene	Protein
***Class I—Inducible nuclear MKPs***								
*DUSP1*	MKP-1					
*DUSP2*	PAC-2					
*DUSP4*	MKP-2					
*DUSP5*	hVH3/B23					
***Class II—Cytoplasmic ERK-specific MKPs***								
*DUSP6*	MKP-3	MKP3	AGAP012237	AgMKP3	ASTE011534	AsMKP3
*DUSP7*	PYST2					
*DUSP9*	MKP-4					
***Class III—Cytoplasmic/nuclear p38- and JNK-specific MKPs***								
*DUSP8*	hVH5					
*DUSP10*	MKP-5	puckered	AGAP004353	AgMKP5	ASTE002907	AsMKP5
*DUSP16*	MKP-7					
***Atypical DUSPs***								
*DUSP12*	VHX	MKP4	AGAP002108	AgMKP4	ASTE004228	AsMKP4
*DUSP13*	BEDPA	CG7378	AGAP008228	—	ASTE005078	—
*DUSP14*	MKP-6	CG15528	AGAP006593	—	ASTE007826	—
*DUSP19*	SKRP1	MKP	AGAP009903	AgMKP	ASTE009886	AsMKP

*Homo sapiens and *Mus musculus*

### Overexpression of MKP4 in *A*. *stephensi* cells inhibited lipopolysaccharide-induced JNK phosphorylation

**Fig 4:**
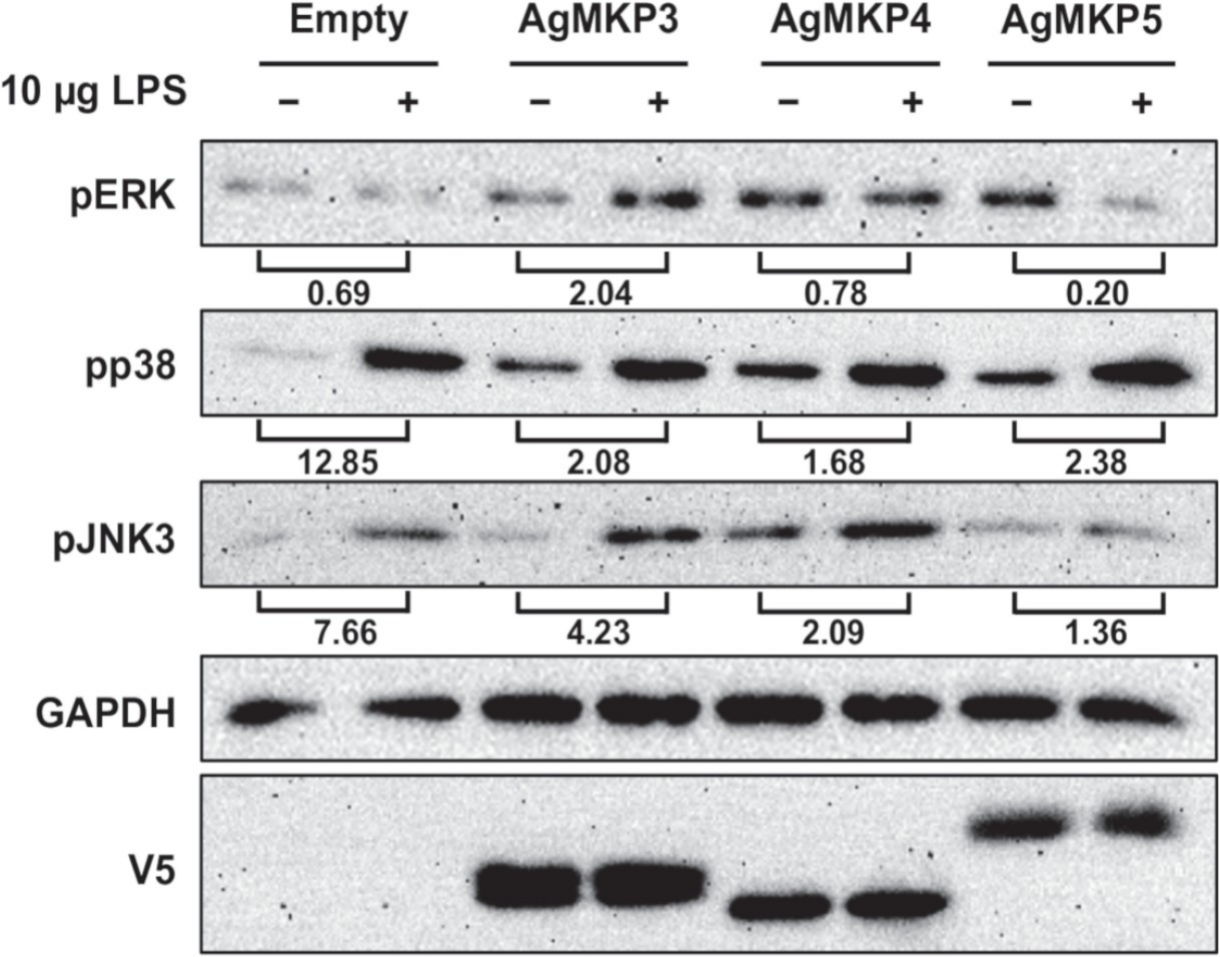
MKP overexpression in ASE cells differentially altered LPS-induced *A*. *stephensi* MAPK phosphorylation. ERK, p38, and JNK3 phosphorylation was detected in response to LPS stimulation in ASE cells transfected with empty plasmid or plasmids overexpressing *A*. *gambiae* MKP3, MKP4, or MKP5. As noted in the text, pJNK1 levels are very low to not detectable in ASE cells. Numbers indicated below the brackets indicate fold changes in MAPK phosphorylation in LPS-treated (+) relative to untreated control (-) cells. Detection of GAPDH was used to assess protein loading and V5 detection confirmed MKP protein overexpression.

To investigate whether mosquito MKPs regulate the activation of MAPKs, ASE cells were transiently transfected with V5-tagged plasmids encoding the complete protein sequence of *A*. *gambiae* MKP3, MKP4, or MKP5, which are 75–85% identical in amino acid sequence to the *A*. *stephensi* orthologs (not shown). Given that *D*. *melanogaster* MKP5 (Puckered) is both activatable and active in human embryonic kidney (HEK293) cells [38], highly conserved patterns of activity among heterologous *Anopheles* MKPs were expected among congeners. At 24 h post-transfection, cells were exposed to 10 μg *E*. *coli* lipopolysaccharide (LPS) for 15 min and cell lysates were assayed by western blotting to measure levels of phosphorylated ERK, JNK, and p38 MAPK [24] (**Fig 4**). In ASE cells, pJNK3 is detectable with anti-pJNK 1/2 antibody, whereas pJNK1 is faint or undetectable for reasons that are unclear to us, so the following observations are presented in this context. In ASE cells transfected with an empty vector, LPS treatment was associated with reduced ERK phosphorylation and increased p38 MAPK and JNK3 phosphorylation (e.g., fold changes of 0.69, 12.85 and 7.66, respectively, as indicated; **Fig 4**). In the presence of MKP3 overexpression, LPS induced higher ERK phosphorylation and lower relative p38 and JNK3 phosphorylation levels than observed in the empty vector controls. With MKP4 overexpression, the effect of LPS on ERK phosphorylation was similar to control, but the relative fold changes in p38 and JNK3 phosphorylation in response to LPS were lower than those observed in the controls. With overexpression of MKP5 (Puckered), relative p38 and JNK3 phosphorylation levels after LPS treatment were also reduced relative to controls, but MKP5 overexpression notably reduced ERK phosphorylation after treatment with LPS. Thus, out of the three phosphatases, MKP4 had the most desired characteristics, with minimal effect on ERK phosphorylation and reductions in inducible p38 MAPK and JNK3 phosphorylation compared to controls. Further, these data are consistent with observations that *D*. *melanogaster* MKP4 negatively regulates JNK activation, but not that of ERK [36].

### Generation and characterization of CP-AsMKP4-HA transgenic *A*. *stephensi*

**Fig 5:**
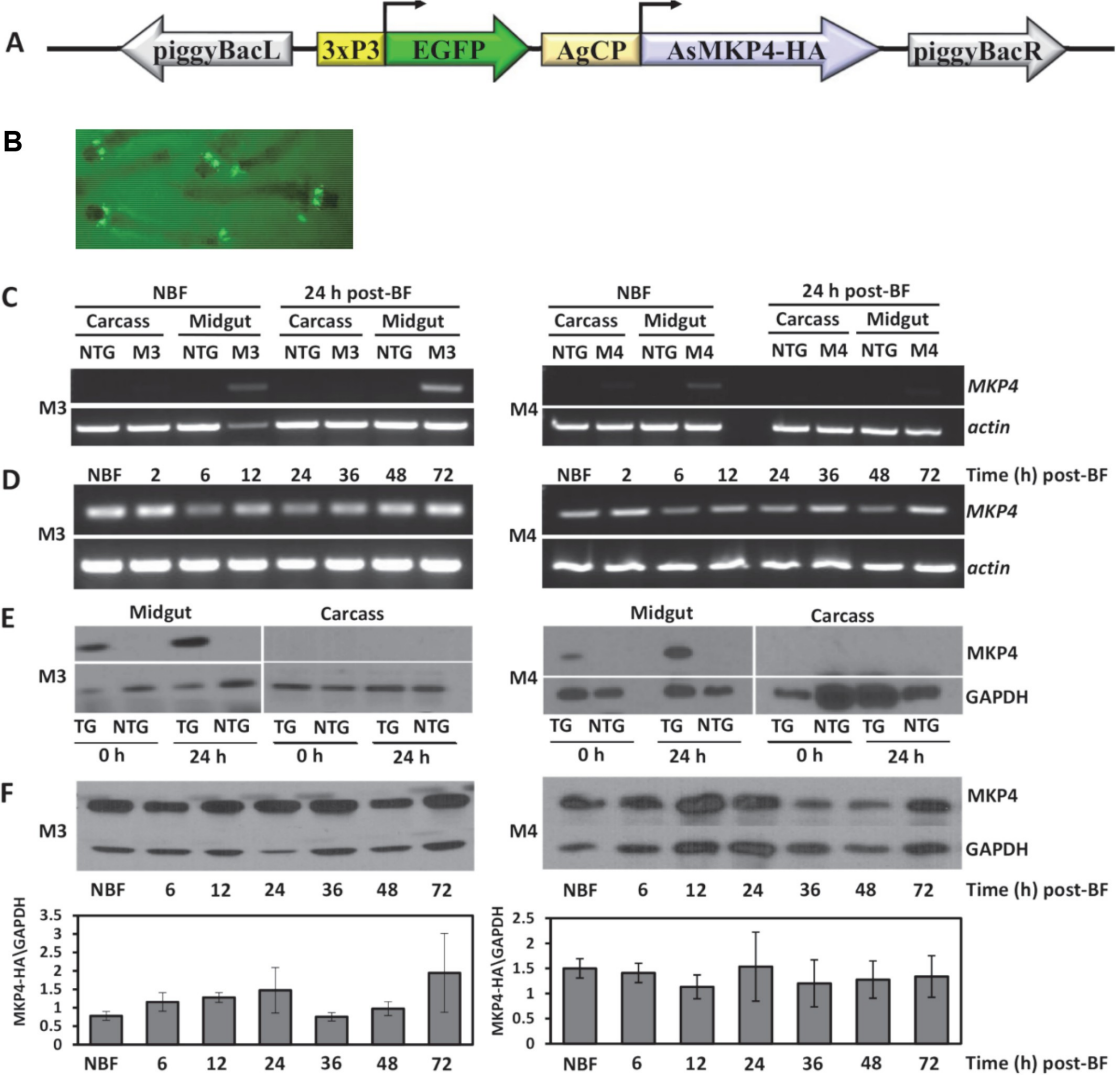
MKP4 transgene transcript and protein expression were midgut-specific and induced by blood feeding in M3 and M4 lines of transgenic female *A*. *stephensi*. **(A)** Diagram of the transformation construct showing the marker gene EGFP driven by the eye-specific 3XP3 promoter and the *A*. *stephensi* MKP4-HA construct driven by the *A*. *gambiae* carboxypeptidase (AgCP) promoter and flanked by *pBac* inverted terminal repeats. **(B)** EGFP fluorescence in MKP4 transgenic *A*. *stephensi* larvae. **(C)** Transcript levels of the *MKP4* transgene and *actin* in M3 and M4 transgenic lines and non-transgenic (NTG) *A*. *stephensi* midgut and carcass (body minus midgut) tissues in non-blood fed (NBF) individuals and at 24 h after blood feeding (BF), and **(D)** in midgut tissue of M3 and M4 transgenic *A*. *stephensi* from 2–72 h post-blood feeding. **(E)** Transgene protein expression levels in M3 and M4 lines of MKP4 transgenic (TG) and non-transgenic (NTG) *A*. *stephensi* midgut and carcass (body minus midgut) tissues before and 24 h after a blood meal. **(F)** Transgene protein expression patterns in non-blood fed (NBF) transgenic and non-transgenic *A*. *stephensi* midgut and carcass and from 6–72 h after blood feeding (BF). Relative MKP4 protein expression levels for all replicates are plotted below. All gene and protein expression assays were replicated with three separate cohorts of mosquitoes.

Based on the effects of MKP4 overexpression in *A*. *stephensi* cells *in vitro* (**Fig 4**), we genetically engineered *A*. *stephensi* to overexpress MKP4 in the midgut epithelium (CP-AsMKP4-HA) to examine regulation of longevity, fecundity, immunity, and *P*. *falciparum* infection. A total of 10 transgenic *A*. *stephensi* lines overexpressing MKP4 under the control of the midgut-specific CP promoter (**Fig 5A and 5B**) were generated. Two lines (M3 and M4) with the highest levels of transgene mRNA and protein expression were selected for further analyses. While both lines had robust transgene expression, the M3 line expressed significantly more MKP4 protein than did the M4 line (**S1 Fig**). Transcript expression of *CP-AsMKP4-HA* was localized to the midgut in both lines and induced by blood feeding in adult female transgenic mosquitoes (**Fig 5C and 5D**). Expression of the CP-AsMKP4-HA protein was similarly detected only in the midgut of both lines and not in the carcass (**Fig 5E**). The M3 line expressed MKP4 protein in a manner typical for transgene expression driven by the carboxypeptidase promoter in *A*. *stephensi*. Specifically, MKP4 protein expression in the M3 line was increased for up to 24 h after the bloodmeal, after which it returned to basal levels (**Fig 5F**). An increase in MKP4 protein was also observed at 72 h but this was highly variable among the replicates. In contrast, MKP4 protein expression in the M4 line did not increase following the bloodmeal, but instead remained consistent with the non-blood fed background level of MKP4 protein expression (**Fig 5F**). It is important to note that expression levels between M3 and M4 cannot be directly compared because independent immunoblots were performed. However, as noted above the M3 line consistently expressed more MKP4 protein than the M4 line (**S1 Fig**). These differences in protein expression between the M3 and M4 lines likely account for why, in the following assays, M3 phenotypes were significantly different from control and largely mirrored JNK inhibitor assays, whereas M4 phenotypes were typically not different from control. Finally, results from sequencing inverse PCR fragments revealed that the transgene did not insert into any known or predicted *A*. *stephensi* genes for either the M3 or M4 lines (**S2 Fig**). Both lines were maintained as hemizygous by outcrossing female mosquitoes in each generation to non-transgenic male *A*. *stephensi*.

### JNK inhibition in the *A*. *stephensi* midgut by SMIs and MKP4 overexpression

**Fig 6:**
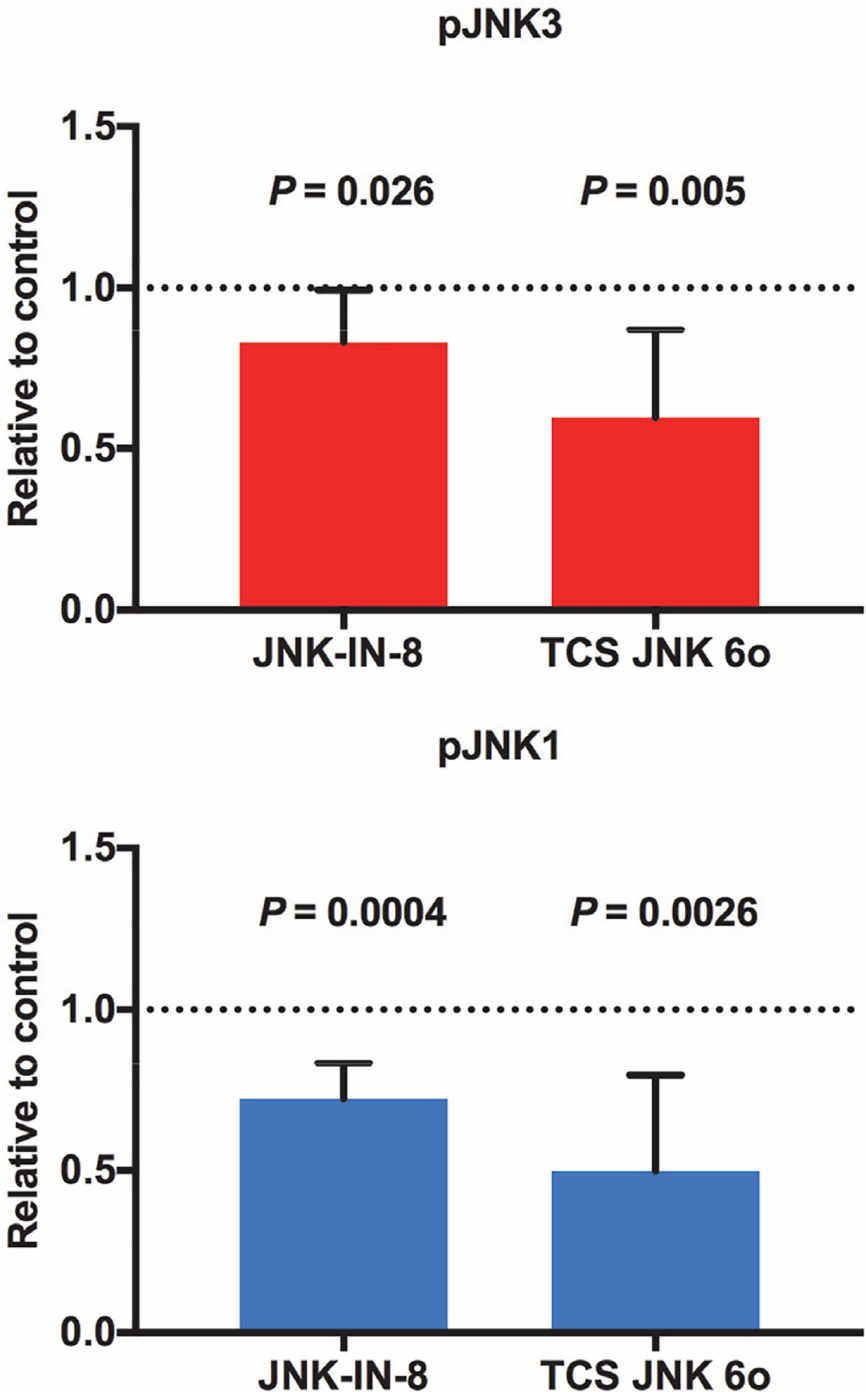
Provision of JNK-IN-8 and TCS JNK 6o in artificial blood meals inhibited JNK1 and JNK3 phosphorylation in the *A*. *stephensi* midgut. Three-day old female *A*. *stephensi* were allowed to feed for 30 min on an uninfected blood meal containing 1 μM JNK-IN-8 or 1 μM TCS JNK 6o or on a blood meal supplemented with an equivalent volume of diluent (DMSO) as a control. A total of 25 midguts from each group were dissected and pooled at 1–3 h post feeding and processed for western blotting. JNK phosphorylation levels were normalized to control (set at 1.0, dotted line). This experiment was replicated three times with separate cohorts of mosquitoes and fold changes in pJNK levels relative to matched controls were analyzed by Student’s t-test.

The highly selective, potent inhibitor JNK-IN-8 forms a covalent bond with a conserved cysteine residue in JNK [39], whereas TCS JNK 6o is an ATP-competitive pan-JNK inhibitor [40]. The residues forming the ATP binding site are largely conserved in *A*. *stephensi* JNK1 and JNK3, but the conserved cysteine is missing in JNK3 (**S3 Fig**). To test the activity of these small molecule inhibitors (SMIs) against JNK signaling in *A*. *stephensi*, 3-day old female *A*. *stephensi* were provided artificial blood meals supplemented with 1 μM JNK-IN-8, 1 μM TCS JNK 6o, or an equivalent volume of diluent (dimethyl sulfoxide, DMSO) added to the blood meal as a control. At 1–3 h post feeding, 25 midguts from each group were dissected, pooled, and processed for western blotting for pJNK. Both SMIs significantly inhibited JNK phosphorylation (**Fig 6**), with differing effects of the two SMIs on JNK3 that might derive from the mechanisms described above. Specifically, midgut pJNK3 and pJNK1 levels in JNK-IN-8-treated mosquitoes were 82.8% and 72% of control levels, respectively, while pJNK3 and pJNK1 levels in TCS JNK 6o-treated mosquitoes were 59.6% and 49.8% of control levels, respectively.

**Fig 7:**
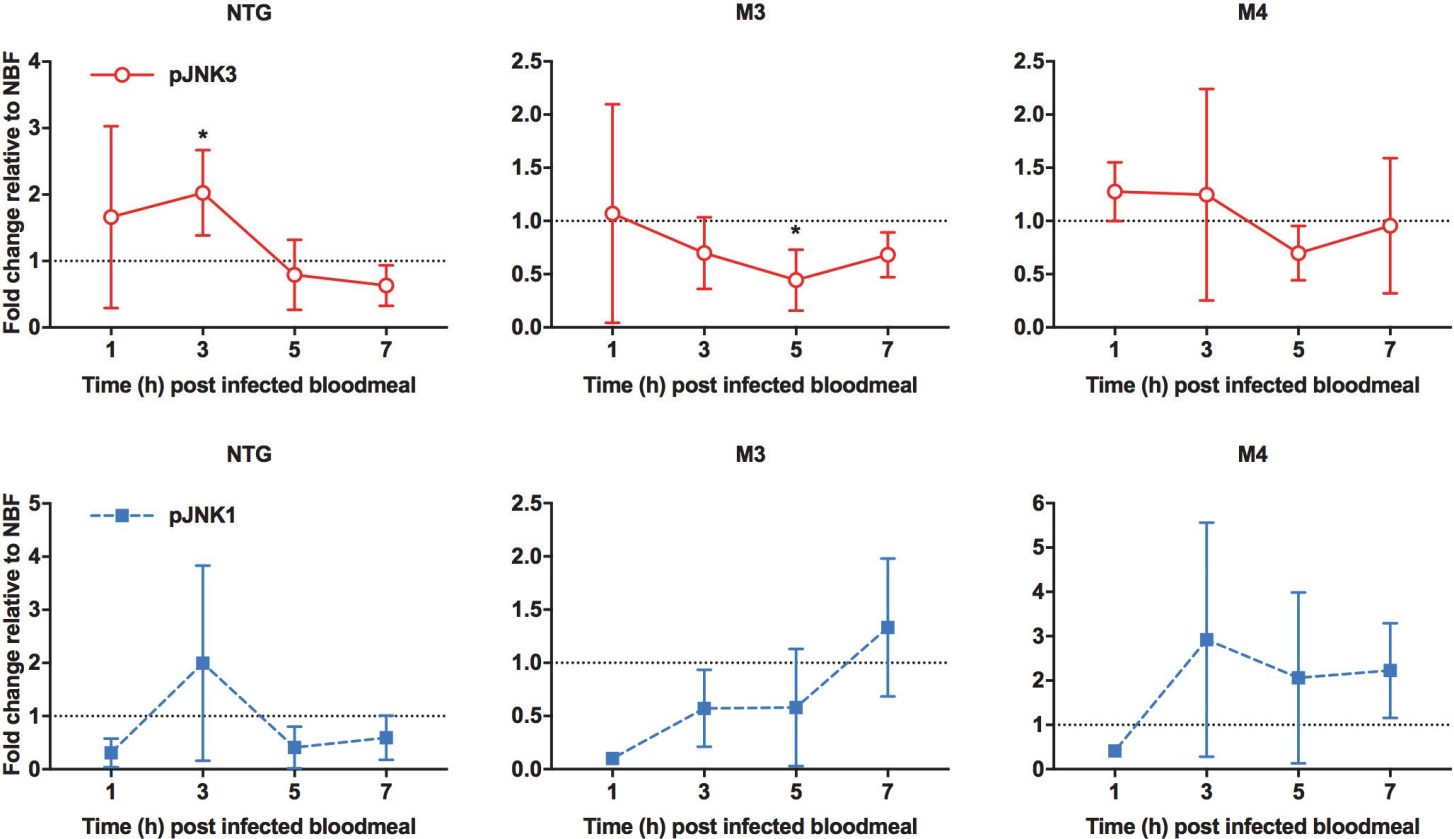
Midgut JNK3 phosphorylation levels were reduced below non-blood fed levels by MKP4 overexpression in *P*. *falciparum*-infected M3 line *A*. *stephensi*. Three-day old M3 and M4 line MKP4 transgenic and non-transgenic (NTG) female *A*. *stephensi* were allowed to feed on *P*. *falciparum-*infected blood meals for 30 min. Total proteins from midguts dissected at 1, 3, 5, and 7 h after feeding were processed for western blotting. pJNK levels were first normalized to GAPDH and then to pJNK in non-blood fed (NBF) mosquitoes within each group (set at 1, black dotted line). These experiments were replicated with 2–5 separate cohorts of mosquitoes. Fold changes relative to NBF controls at each timepoint were analyzed by Student’s t-test (*, *P* < 0.05).

To determine whether MKP4 overexpression in the *A*. *stephensi* midgut could inhibit inducible JNK phosphorylation, we provided identical *P*. *falciparum*-infected blood meals to M3 and M4 transgenic lines and to non-transgenic controls and assessed pJNK levels in these insects relative to non-fed matched controls. This normalization was selected to allow us to determine whether MKP4 overexpression would be sufficient to alter pJNK signaling induced not only by infection, but also by ingestion of blood alone (**Fig 2**). In non-transgenic mosquitoes, JNK3 phosphorylation levels were significantly increased above non-fed control levels (dotted line) by 3 h post-feeding before returning to this baseline (**Fig 7**). In contrast, JNK3 phosphorylation declined significantly by 5 h post-feeding in M3 transgenic mosquitoes (**Fig 7**), consistent with MKP4 repression of inducible JNK3 phosphorylation in ASE cells (**Fig 4**), whereas JNK3 phosphorylation was not different from control levels at any time post-blood feeding in M4 transgenic mosquitoes. JNK1 phosphorylation levels were not different from control levels in non-transgenic or transgenic mosquitoes at any time post-blood feeding, although pJNK1 levels trended downward post-blood feeding relative to non-fed control levels only in the M3 transgenic line (**Fig 7**). Additionally, as with MKP4 overexpression in ASE cells, M3 transgenic mosquitoes overexpressing MKP4 in the midgut did not display increased p38 phosphorylation **(S4A Fig)** and ERK phosphorylation was unaffected **(S4B Fig)** following a *P*. *falciparum*-infected bloodmeal relative to non-transgenic controls.

### The impacts of SMIs and MKP4 midgut overexpression on *A*. *stephensi* fitness

**Fig 8:**
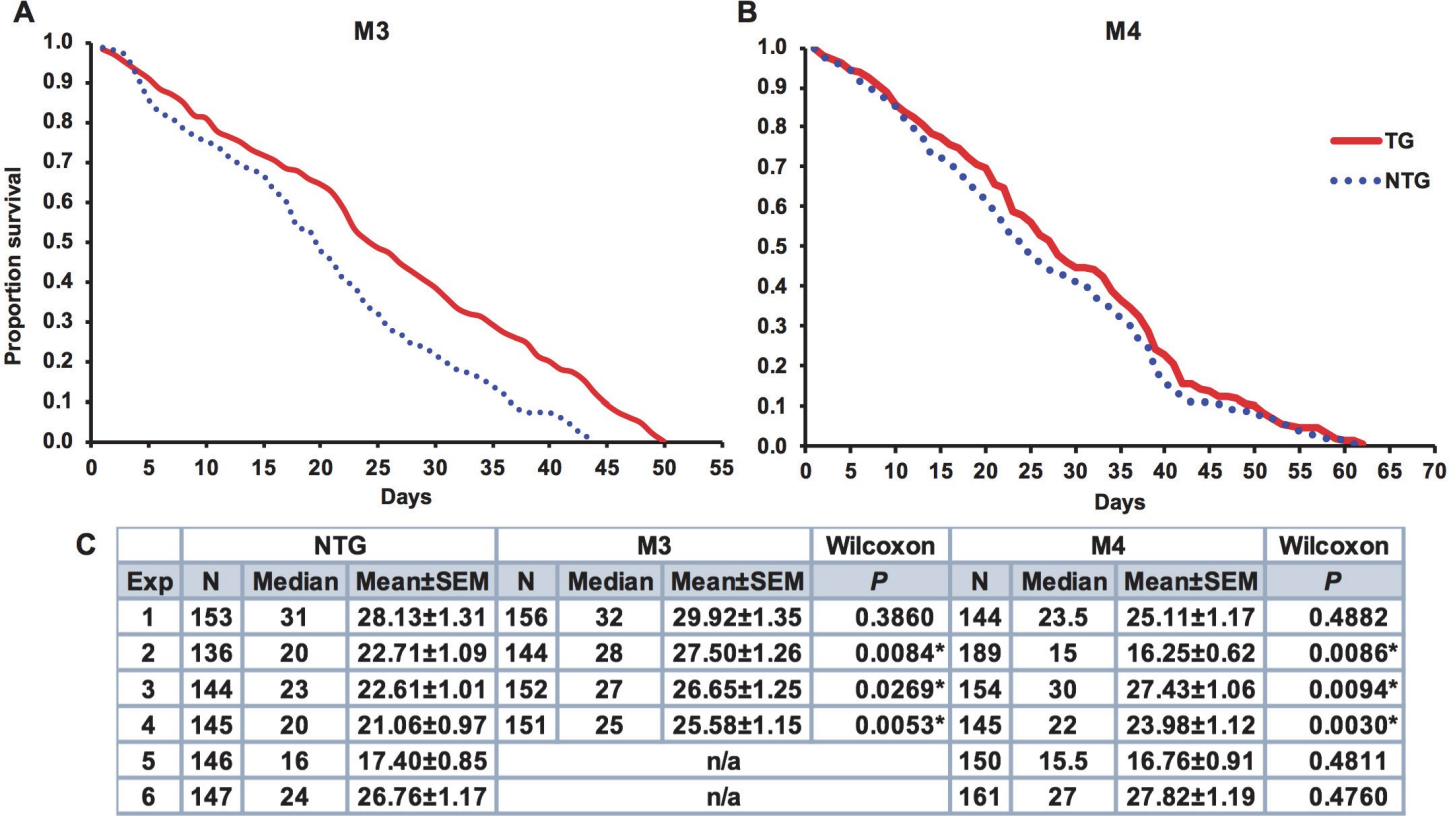
MKP4 overexpression in the midgut of *A*. *stephensi* increased median lifespan in M3 line transgenic relative to non-transgenic mosquitoes. Representative survivorship curves for M3 line MKP4 transgenic **(A)** and M4 line MKP4 transgenic **(B)** *A*. *stephensi* relative to non-transgenic (NTG) controls. Lifespan experiments were replicated four (M3) or six (M4) times with separate cohorts of mosquitoes. **(C)** Summary of sample sizes, medians, means, and significance relative to NTG mosquitoes as determined by Gehan-Breslow-Wilcoxon analysis within individual experiments. Meta-analysis of the replicates revealed no difference in median lifespans between M4 line and NTG females, but median lifespan of the M3 line was significantly longer than that of NTG females (one-way ANOVA, P = 0.05).

Based on previous observations from *D*. *melanogaster*, we hypothesized that exposure to JNK SMIs in weekly blood meals should extend mosquito lifespan relative to controls [28, 41]. However, an analysis of median lifespans in 11 separate cohorts of *A*. *stephensi* provided weekly blood meals supplemented with 1 μM JNK-IN-8 or 1 μM TCS JNK 6o indicated that treated mosquitoes were not significantly different from matched controls that received blood meals supplemented with an equivalent volume of diluent (one-way ANOVA, P = 0.80; **Table 2**). Accordingly, we speculated that exposure to JNK SMIs once per week was not sufficient to impact lifespan, but that perhaps sustained JNK inhibition could impact longevity. To test this, female MKP4 transgenic mosquitoes were provided blood meals three times a week to induce consistent overexpression of the *MKP4* transgene in the midgut epithelium. Here, blood feeding extended median lifespan in three of four cohorts of M3 mosquitoes by an average of 5.7 days (**Fig 8A and 8C**), but significantly reduced median lifespan in one M4 cohort and enhanced median lifespan by 4.5 days in only two out of six M4 cohorts (**Fig 8B and 8C**). An analysis of these replicates indicated that there was no difference in median lifespan between M4 line and non-transgenic females, but median lifespan of the M3 line was significantly longer than that of non-transgenic females (one-way ANOVA, P = 0.05).

**Table 2:** Analysis of replicate lifespan studies of *A*. *stephensi* provided weekly blood meals with JNK-IN-8 or TCS JNK 6o relative to controls.

Replicate		Log-Rank Mantel Cox	Significantly different?	Gehan-Breslow-Wilcoxon	Significantly different?
1	Ctrl vs. IN8	0.0002	Y	0.0005	Y
	Ctrl vs. 6o	0.0060	Y	0.0066	Y
2	Ctrl vs. IN8	0.2258	N	0.918	N
	Ctrl vs. 6o	0.2920	N	0.7092	N
3	Ctrl vs. IN8	0.2557	N	0.4146	N
	Ctrl vs. 6o	0.0840	N	0.5354	N
4	Ctrl vs. IN8	0.6492	N	0.9208	N
	Ctrl vs. 6o	0.7425	N	0.8301	N
5	Ctrl vs. IN8	0.8337	N	0.7483	N
	Ctrl vs. 6o	0.0132	Y	0.0217	Y
6	Ctrl vs. IN8	0.8936	N	0.6031	N
	Ctrl vs. 6o	0.7193	N	0.4041	N
7	Ctrl vs. IN8	0.0038	Y	0.1094	N
	Ctrl vs. 6o	0.9906	N	0.5927	N
8	Ctrl vs. IN8	<0.0001	Y	<0.0001	Y
	Ctrl vs. 6o	0.0408	Y	0.221	N
9	Ctrl vs. IN8	0.9591	N	0.6036	N
	Ctrl vs. 6o	0.1263	N	0.2406	N
10	Ctrl vs. IN8	0.0042	Y	0.0226	Y
	Ctrl vs. 6o	0.0644	N	0.0566	N
11	Ctrl vs. IN8	0.0967	N	0.9709	N
	Ctrl vs. 6o	0.0204	Y	0.1332	N

**Fig 9:**
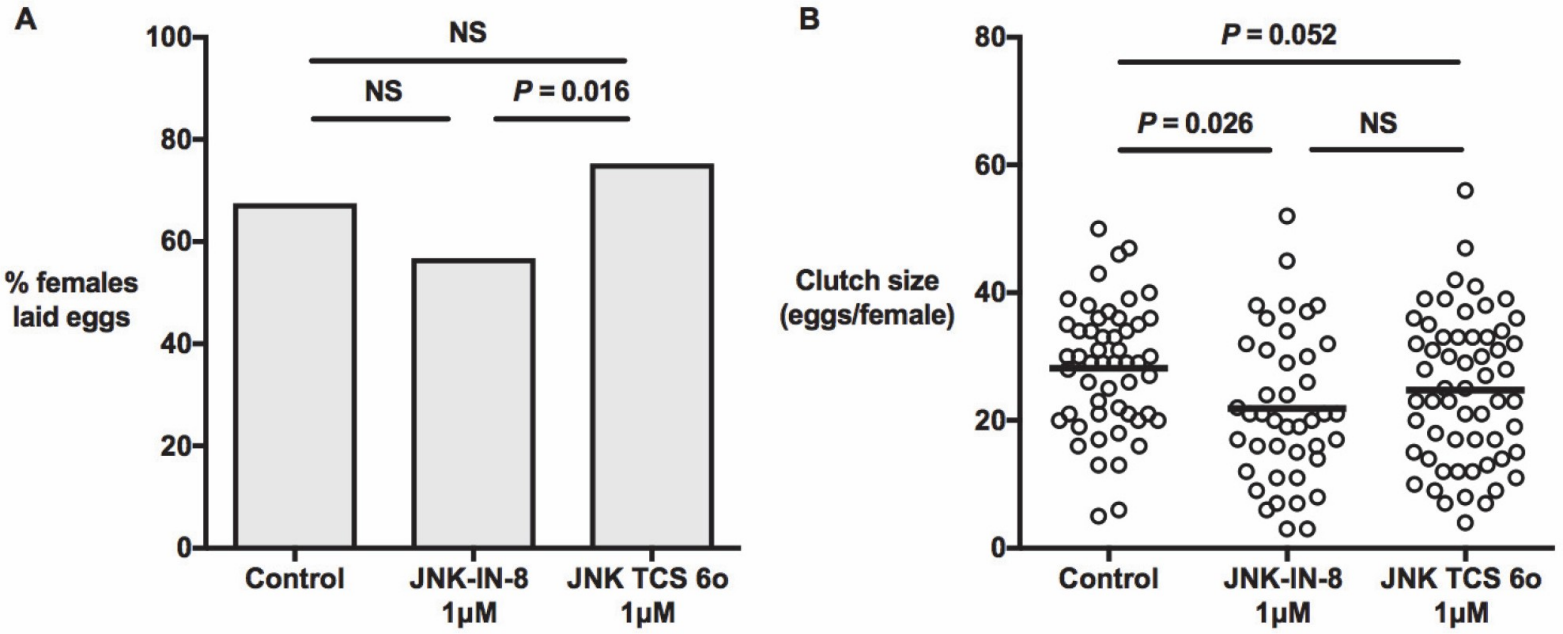
Provision of JNK-IN-8 and TCS JNK 6o in artificial blood meals reduced eggs laid per female *A*. *stephensi* during the first gonotrophic cycle. Three-day old *A*. *stephensi* were provided blood meals supplemented with 1 μM JNK-IN-8, TCS JNK 6o, or blood supplemented with an equivalent volume of diluent as a control. At 24 h post feeding, females were housed individually in modified 50 ml conical tubes and provided water for oviposition for two days. Eggs were washed onto filter paper, photographed, and counted using ImageJ software. The experiment was replicated four times with separate cohorts of mosquitoes. (**A**) Proportions of females that laid at least one egg were analyzed by Chi-square test. (**B**) Clutch sizes relative to controls were analyzed by Mann-Whitney test (NS = not significant).

In separate studies, we examined the effects of JNK SMIs and MKP4 overexpression on reproductive output. The proportions of *A*. *stephensi* females that laid eggs during the first gonotrophic cycle after feeding on blood meals containing 1 μM JNK-IN-8 or 1 μM TCS JNK 6o were not significantly different from female mosquitoes that received a blood meal supplemented with an equivalent volume of diluent as a control (**Fig 9A**). However, the average clutch sizes from groups that received either JNK inhibitor were lower than the control group (**Fig 9B**). Specifically, control mosquitoes laid an average of 28.1 eggs per female compared to 21.9 and 24.8 eggs per female in mosquitoes fed JNK-IN-8 (*P* = 0.026) or TCS JNK 6o (*P* = 0.052), respectively (**Fig 9B**), with no difference between the treatment groups.

**Fig 10:**
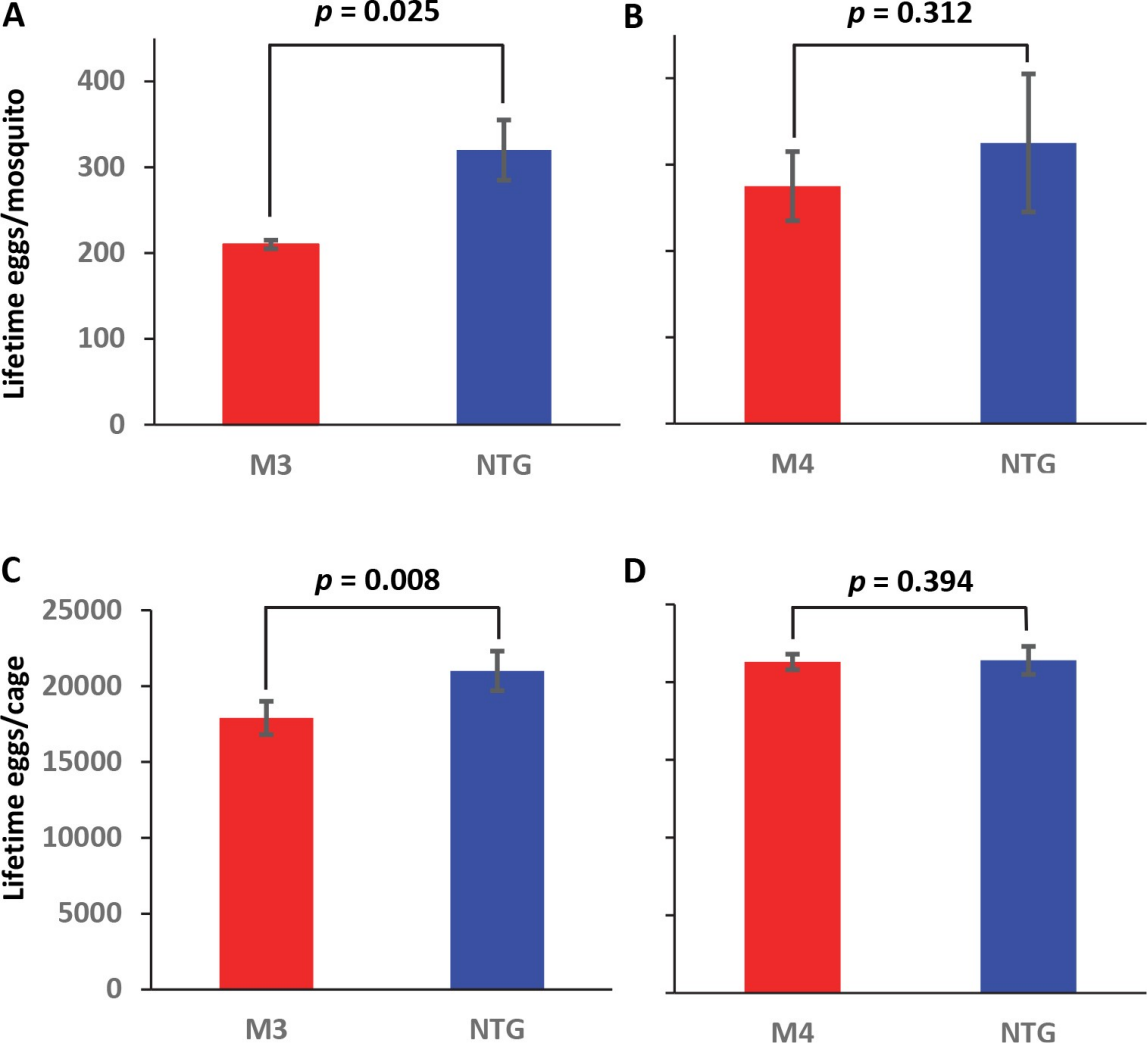
MKP4 overexpression in the midgut of *A*. *stephensi* reduced lifetime fecundity in M3 line transgenic mosquitoes relative to non-transgenic mosquitoes. Lifetime fecundity was assessed at the individual (**A, B**) and cage or population (**C, D**) levels. Bars represent the combined average total number of eggs per individual (**A, B**) or for the entire cage or population (**C, D**) through to the end of lifespan. The experiment was replicated four times with separate cohorts of mosquitoes and data were analyzed using Student’s t-test.

Lifetime fecundity of MKP4 transgenic and non-transgenic *A*. *stephensi* was measured by tallying the total number of eggs produced during female reproductive lifetime. M3 mosquitoes produced significantly fewer eggs per female relative to non-transgenic controls (**Fig 10A**), whereas M4 female output did not differ from controls (**Fig 10B**). M3 transgenic mosquitoes produced fewer eggs relative to non-transgenic females over their reproductive lifespans (**Fig 10C**). In contrast, the M4 transgenic line did not differ significantly from non-transgenic controls (**Fig 10D**). Thus, even with the increased survivorship of M3 transgenic *A*. *stephensi* relative to non-transgenic controls (**Fig 8A and 8C**), the M3 line produced fewer eggs suggesting a physiological trade-off between lifespan extension and reproduction.

### The impact of JNK SMIs and MKP4 overexpression on *P*. *falciparum* infection in *A*. *stephensi*

Three-day old adult female *A*. *stephensi* mosquitoes were provided *P*. *falciparum* gametocyte-enriched blood meals supplemented with 1 μM JNK-IN-8 or 1 μM TCS JNK 6o or an identical blood meal supplemented with an equivalent volume of diluent (DMSO) as a control. At day 10 post-blood feeding, midguts were dissected and stained and the numbers of oocysts were directly counted. In all three replicates, mosquitoes fed an infectious blood meal with JNK-IN-8 had significantly fewer oocysts per midgut compared to control (**Table 3**). Mosquitoes given an infectious blood meal supplemented with TCS JNK 6o had significantly fewer oocysts relative to control in one of three replicates with downward trends in the other two replicates (**Table 3**). Prevalences of infection (i.e., the number of mosquitoes that had at least one oocyst) were consistently and significantly lower in both JNK-IN-8- and TCS JNK 6o-treated females relative to controls as determined by Chi-square analysis (Exp 1, χ^2^ = 20.78, df = 2, *P* = 0.000031; Exp 2, χ^2^ = 11.53, df = 2, *P* = 0.0031; Exp 3, χ^2^ = 24.85, df = 2, *P* < 0.00001).

**Table 3:** Moderate inhibition of JNK reduces *P*. *falciparum* oocyst development in *A*. *stephensi*.

	Control			JNK-IN-8				TCS JNK 6o			
Exp	N	mean oocyst±SEM	% inf	N	mean oocyst±SEM	% inf	p-value	N	mean oocyst±SEM	% inf	p-value
1	42	0.71±0.13	52.4	38	0.34±0.12	21.1	0.0097	38	0.39±0.12	36.3	0.0366
2	38	1.11±0.20	57.9	42	0.43±0.10	35.7	0.0114	41	0.56±0.13	39.0	0.0574
3	46	1.20±0.18	67.4	41	0.54±0.14	31.7	0.0032	44	0.73±0.15	45.5	0.0666

While our data suggested that JNK SMIs alter parasite development in *A*. *stephensi* through effects on mosquito cell signaling, we also examined the possibility that these inhibitors could target parasite viability directly. The *P*. *falciparum* genome encodes two MAPKs, Pfmap-1 and Pfmap-2, that do not cluster phylogenetically with typical MAPKs. In particular, Pfmap-1 clusters with human ERK8 and Pfmap-2 is an atypical MAPK with no homology to ERK1/2, JNK, and p38 MAPK proteins [42]. Nevertheless, we tested the effects of JNK SMIs on parasite asexual growth *in vitro*, a reasonable approach in the absence of a complete sexual stage culture system. Synchronized asexual *P*. *falciparum* parasites were treated with 0.1–10 μM JNK-IN-8 or TCS JNK 6o *in vitro* and their growth was evaluated at 48 and 96 h post treatment. Only when parasites were grown in the presence of high concentrations (10 μM) of JNK-IN-8 were negative effects on asexual growth observed (**S5 Fig**). Importantly, treatment with 1 μM JNK-IN-8 or 1 μM TCS JNK 6o, concentrations used in infectious blood meals for mosquitoes (**Fig 9**), had no significant effects on parasite growth *in vitro* relative to control. Therefore, we inferred that reductions in infection prevalence and intensity due to JNK SMI treatment were due to the effects on mosquito cell signaling and physiology and not due to direct effects of these inhibitors on parasite viability.

**Fig 11:**
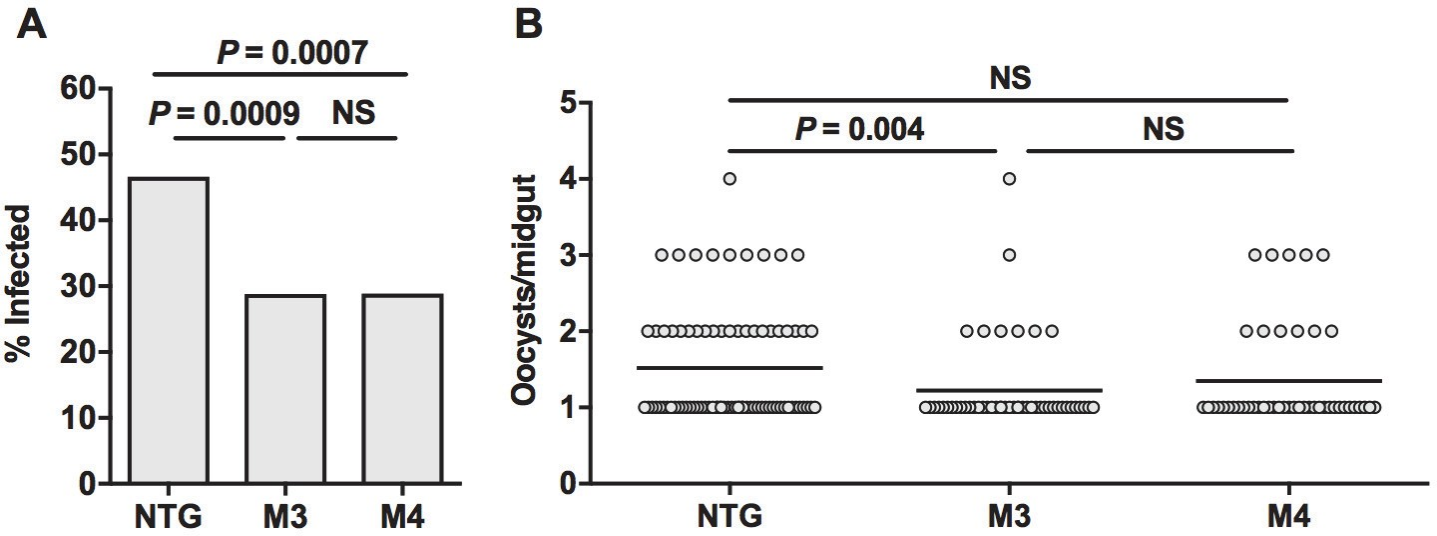
MKP4 overexpression in the midgut of *A*. *stephensi* reduced *P*. *falciparum* infection prevalence and intensity relative to non-transgenic mosquitoes. Data are represented as percentages of mosquitoes infected (infection prevalence, **A**) and mean numbers of oocysts in mosquitoes with at least one midgut oocyst (infection intensity, **B**). Three-day old M3 and M4 line transgenic and non-transgenic (NTG) female *A*. *stephensi* were allowed to feed on *P*. *falciparum-*infected blood meals. Mosquitoes that did not feed or were not fully engorged were removed from the experiment. Ten days after infection, midguts were dissected and the number of *P*. *falciparum* oocysts counted. The experiment was replicated four times with separate cohorts of mosquitoes. Infection intensity data were analyzed using Kruskal-Wallis and Dunn’s post-test. Infection prevalences were analyzed by Chi-square test (NS = not significant).

Malaria parasite infection was also assessed in the M3 and M4 MKP4 transgenic lines. In both lines, MKP4 overexpression in the midgut epithelium significantly reduced the percentage of mosquitoes infected with *P*. *falciparum* relative to non-transgenic *A*. *stephensi* (**Fig 11**). The percentage of mosquitoes with one or more oocysts decreased from an average of 46.6% in non-transgenic *A*. *stephensi* to 28.8% in M3 females and 28.9% in M4 females (**Fig 11A**). Infection intensity, however, was only significantly reduced in M3 females relative to non-transgenic *A*. *stephensi* (**Fig 11B**).

### Effects of JNK SMIs and MKP4 overexpression on anti-parasite gene expression in the *A*. *stephensi* midgut

**Fig 12:**
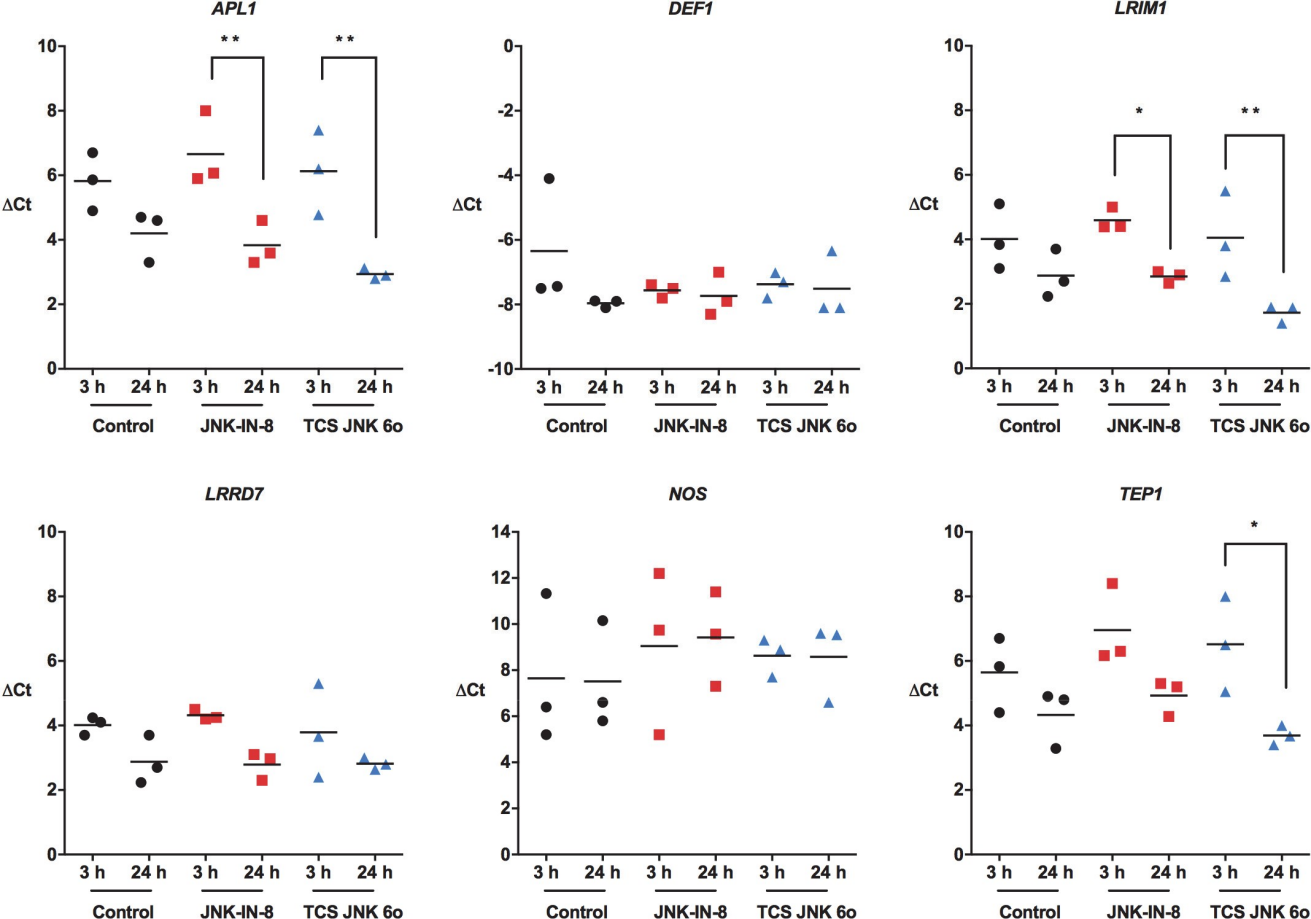
Provision of JNK-IN-8 and TCS JNK 6o in artificial blood meals reduced midgut anti-parasite gene expression in *P*. *falciparum*-infected *A*. *stephensi*. Three-day old *A*. *stephensi* were provided blood meals containing *P*. *falciparum*-infected red blood cells supplemented with 1 μM JNK-IN-8 or 1 μM TCS JNK 6o or an identical blood meal supplemented with an equivalent volume of diluent as a control. At 3 and 24 h post-feeding, midguts were dissected for analyses of anti-parasite gene expression. These assays were replicated three times with separate cohorts of mosquitoes. Data for individual cohorts (circles, squares, triangles) are represented as transcript levels normalized to ribosomal protein *S7* transcript levels and were analyzed by one-way ANOVA (* *P* ≤ 0.05, ** *P* ≤ 0.001).

The JNK signaling pathway is essential for the production of antimicrobial peptides [43, 44], the induction of *NOS* and production of NO [45, 46], and activation of the complement-like system [26]. Thus, we measured midgut expression levels of anti-parasite immune genes involved in these processes following blood meals containing *P*. *falciparum* gametocytes supplemented with 1 μM JNK-IN-8 or 1 μM TCS JNK 6o or an identical blood meal supplemented with an equivalent volume of diluent as a control. In control mosquitoes, transcript levels remained unchanged between 3 and 24 h (**Fig 12**). However, when mosquitoes were treated with 1 μM JNK-IN-8, transcript levels for *APL1* and *LRIM1* were significantly reduced from 3 h to 24 h, while treatment with 1 μM TCS JNK 6o reduced transcript levels for *APL1*, *LRIM1*, and *TEP1* from 3 h to 24 h after feeding (**Fig 12**).

**Fig 13:**
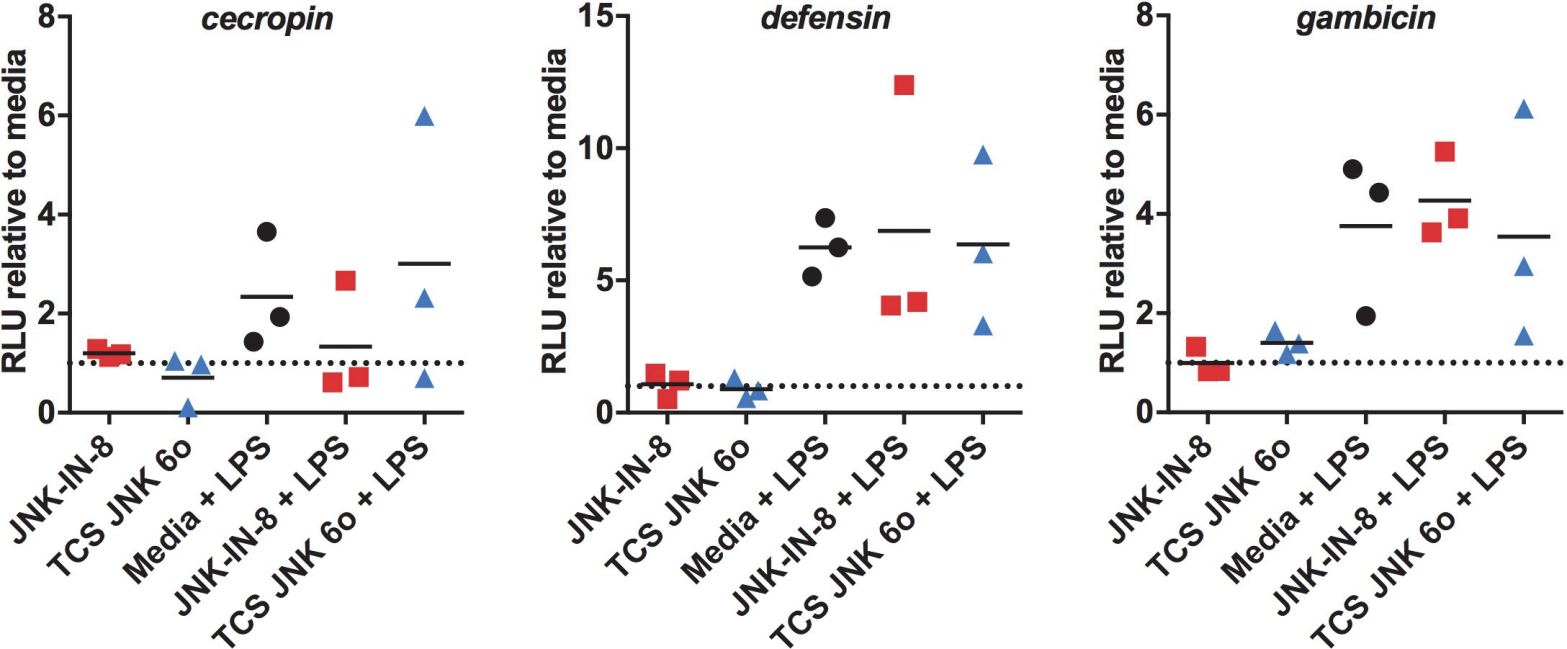
Treatment of ASE cells with JNK-IN-8 or TCS JNK 6o did not alter LPS-induced NF-κB signaling. ASE cells transfected with *cecropin*, *defensin*, or *gambicin* luciferase promoter-reporter plasmid constructs were treated with 1 μM JNK-IN-8, 1 μM TCS JNK 6o, or mock-treated for 1 h prior to stimulation with 100 μg/ml LPS. Data are represented as luciferase activity (relative light units, RLU) normalized to media treated controls (set at 1, dotted line) for each of three replicates (squares, triangles, circles). Treatment with JNK-IN-8 or TCS JNK 6o alone had no effect on NF-κB signaling relative to media control. Further, neither of the JNK SMI + LPS treatments was different from media + LPS by Student’s t-test.

*APL1*, *LRIM1*, and *TEP1* are transcriptionally controlled by Rel2 [9] and Rel1 [47], NF-κB transcription factors that function downstream of Toll and Immune Deficiency (IMD) signaling, respectively. The IMD pathway is also networked with TAK1, a MAP3K that is an upstream activator for JNK. The Rel2 orthologue in *Drosophila*, Relish, modulates the duration of JNK signaling and output in response to Gram-negative infections by inducing the proteasomal degradation of TAK1 [48]. Accordingly, we utilized NF-κB-dependent promoter-luciferase reporter constructs to investigate the extent of crosstalk between NF-κB and JNK signaling in the context of JNK inhibition in *A*. *stephensi* cells. ASE cells were pre-treated with 1 μM JNK-IN-8, 1 μM TCS JNK 6o, or mock-treated for 1 h and then stimulated with LPS prior to assay of *cecropin*, *defensin*, and *gambicin* promoter-reporter activities. LPS induction of promoter activity was expected [12, 49] and observed (**Fig 13**). However, inhibitor pre-treatment prior to LPS stimulation had no effect on NF-κB-dependent promoter activities (**Fig 13**), generally affirming effects of JNK SMIs on innate responses *in vivo* (**Fig 12**).

**Fig 14:**
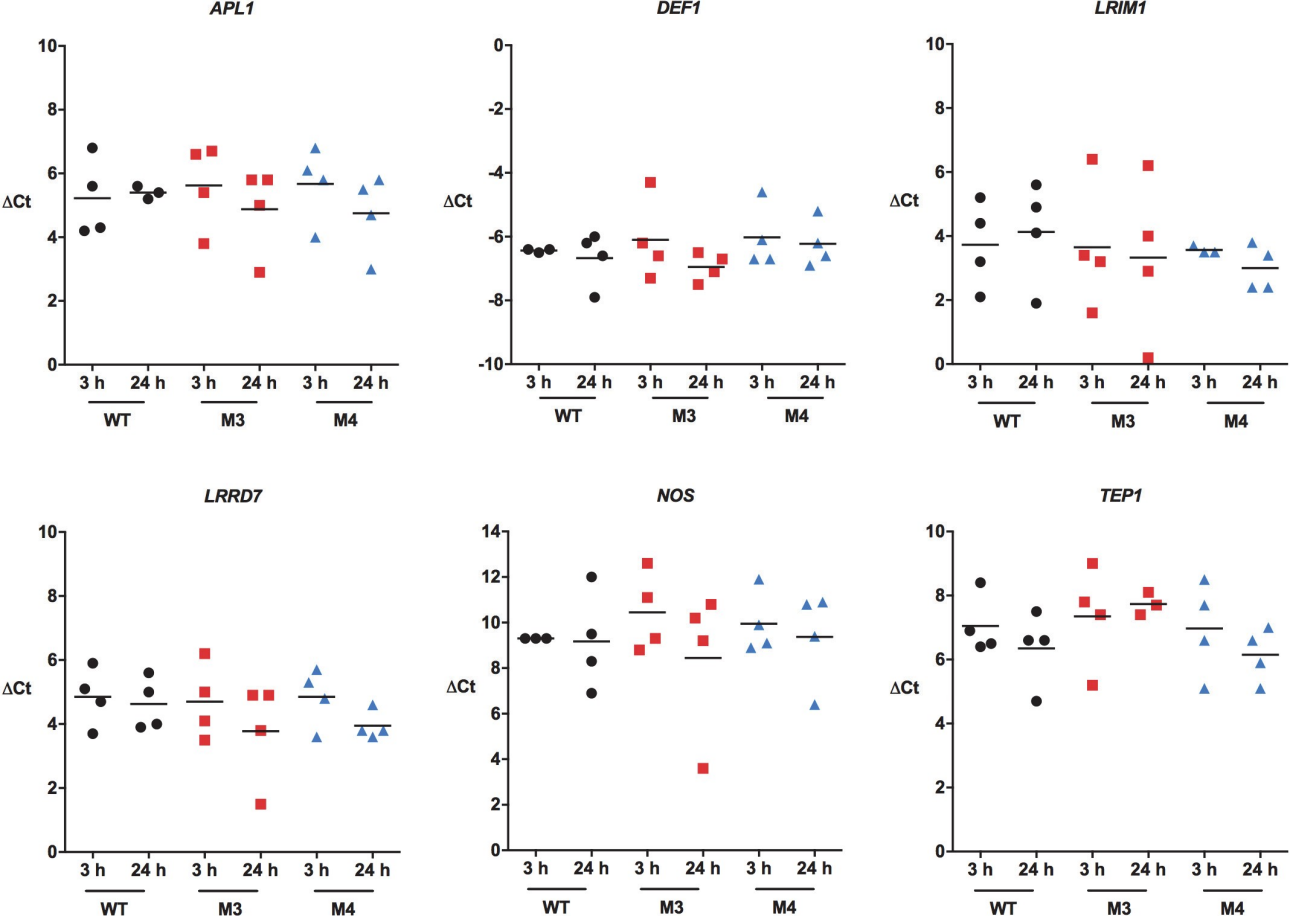
MKP4 overexpression in the midgut of *A*. *stephensi* had no effect on anti-parasite immune gene expression in this tissue. Transgenic and non-transgenic *A*. *stephensi* were allowed to feed on *P*. *falciparum-*infected blood meals. Midguts were dissected at 3 h and 24 h and processed as in Fig 15. The experiment was replicated four times with separate cohorts of mosquitoes. Data are represented as transcript levels normalized to the mosquito housekeeping gene ribosomal protein *S7*. Data were analyzed by one-way ANOVA.

We also quantified midgut anti-parasite gene expression in M3 and M4 MKP transgenic *A*. *stephensi* females and in non-transgenic controls fed identical *P*. *falciparum*-infected blood meals. Notably, there were no significant differences in transcript levels between 3 h and 24 h post-infection in either M3 or M4 mosquitoes (**Fig 14**). While MKP4 inhibited inducible phosphorylation of both JNK and p38 MAPK (**Figs 4 and S4**), our previous studies showed that specific inhibition of p38 MAPK *increased* midgut expression of *LRIM1*, *LRRD7*, *NOS*, *TEP1*, and *APL1* [12], indicating that the effects of MKP4 overexpression are attributable to inhibition of JNK signaling, but not p38 MAPK signaling, *in vivo* in *A*. *stephensi*. Collectively, these observations and our *in vivo* (**Fig 12**) and *in vitro* data for JNK SMIs (**Fig 13**) suggested that transcriptional regulation of anti-parasite genes does not account for observed reductions in *P*. *falciparum* development in *A*. *stephensi* associated with JNK inhibition.

### JNK inhibition did not enhance the *A*. *stephensi* midgut epithelial barrier

**Fig 15:**
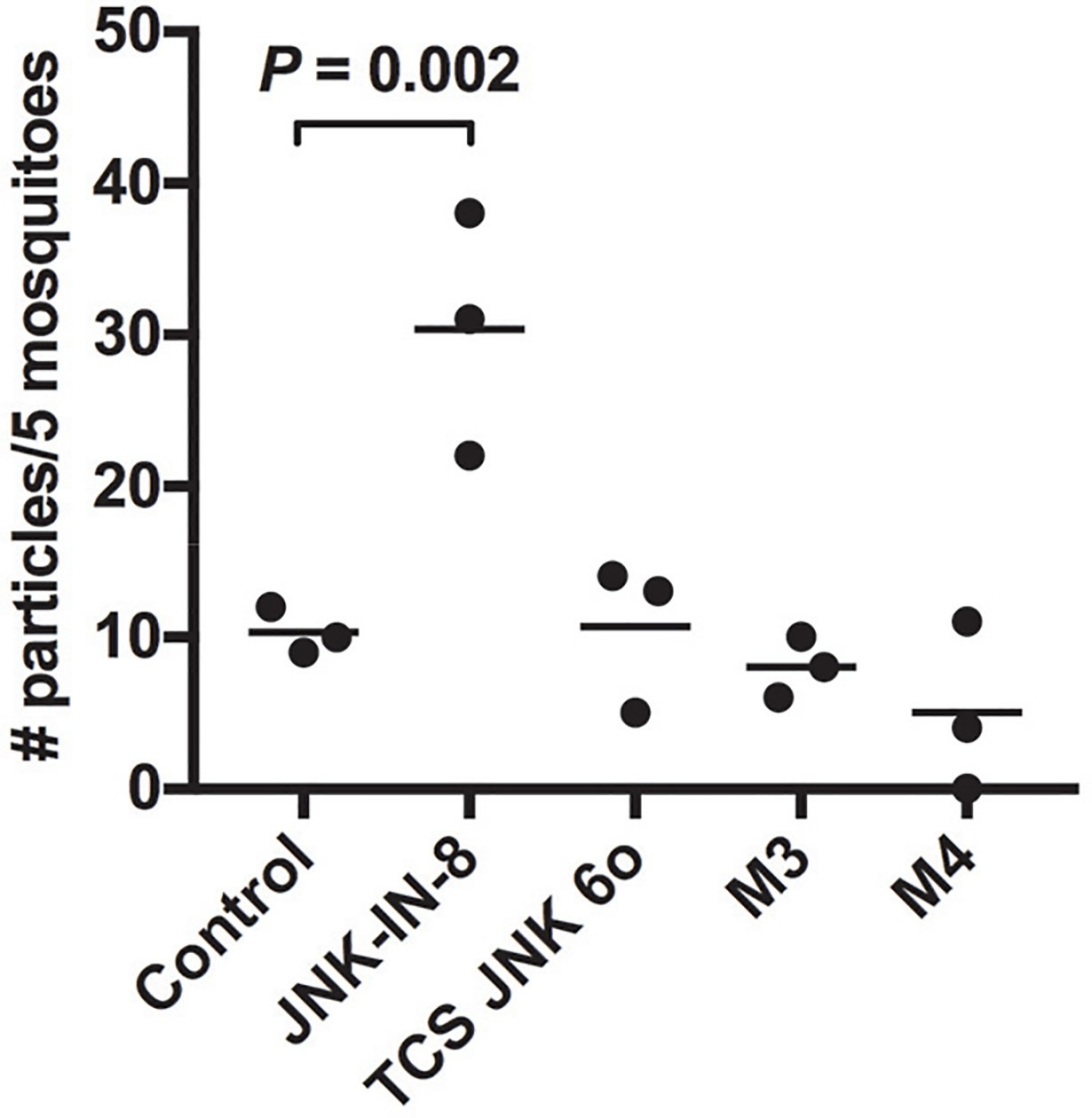
Midgut permeability was inconsistently altered by treatment with JNK SMIs and unaffected by midgut MKP4 overexpression. Non-transgenic (control) *A*. *stephensi* (3–5 day old) were fed on artificial blood meals containing 2.0–2.4 μm magnetic fluorescent particles (Spherotech) with or without supplementation with 1 μM JNK-IN-8 or 1 μM TCS JNK 6o. Particle numbers were quantified at 72 h post-blood feeding as described in the methods. Dots represent particle numbers per five mosquitoes and bars indicate the means. Data were analyzed by one-way ANOVA.

Although JNK inhibition was not associated with enhanced NF-κB-mediated anti-parasite defenses in *A*. *stephensi*, studies in *D*. *melanogaster* suggested that other JNK-specific alterations to the midgut might be associated with enhanced resistance. Specifically, chronic JNK signaling in aged flies can lead to loss of tissue homeostasis but moderate inhibition of JNK reduced age-related midgut dysplasia and extended lifespan [50]. We previously reported that resistance to *P*. *falciparum* infection in *A*. *stephensi* can be associated with increased midgut epithelial integrity [14, 16, 49], suggesting that JNK SMIs might enhance resistance by increasing midgut integrity. To test this, we modified a previous protocol to test midgut permeability with blood meals containing fluorescent particles [14] but without parasites to exclude confounding effects on the midgut epithelium related to parasite infection and invasion. Relative to controls, the midgut was significantly more permeable in mosquitoes fed blood meals supplemented with 1 μM JNK-IN-8, whereas there was no effect of TCS JNK 6o (**Fig 15**), suggesting that JNK SMIs do not enhance resistance by consistently improving midgut barrier integrity. To examine this further, we tested midgut barrier integrity in MKP4 transgenic mosquitoes with the same protocol. Here, MKP4 overexpression did not alter midgut permeability in either transgenic line relative to control (**Fig 15**), again suggesting that JNK inhibition does not enhance resistance to *P*. *falciparum* infection by enhancing midgut barrier integrity.

### Effects of JNK inhibition on the *A*. *stephensi* midgut metabolome

We previously used metabolomics to define the nature of midgut-associated insulin-like peptide (ILP) regulation of *P*. *falciparum* resistance in *A*. *stephensi* [22], so this approach was chosen to help us define the nature of parasite resistance associated with JNK inhibition. While our data suggested that MKP4 activity is likely specific to JNK signaling in the *A*. *stephensi* midgut, we could not exclude the potential for MKP4 effects on p38 MAPK signaling (**Figs 4 and S4**), so we analyzed the effects of JNK SMIs on the metabolome of the *A*. *stephensi* midgut epithelium to more clearly define the effects of JNK inhibition.

**Fig 16:**
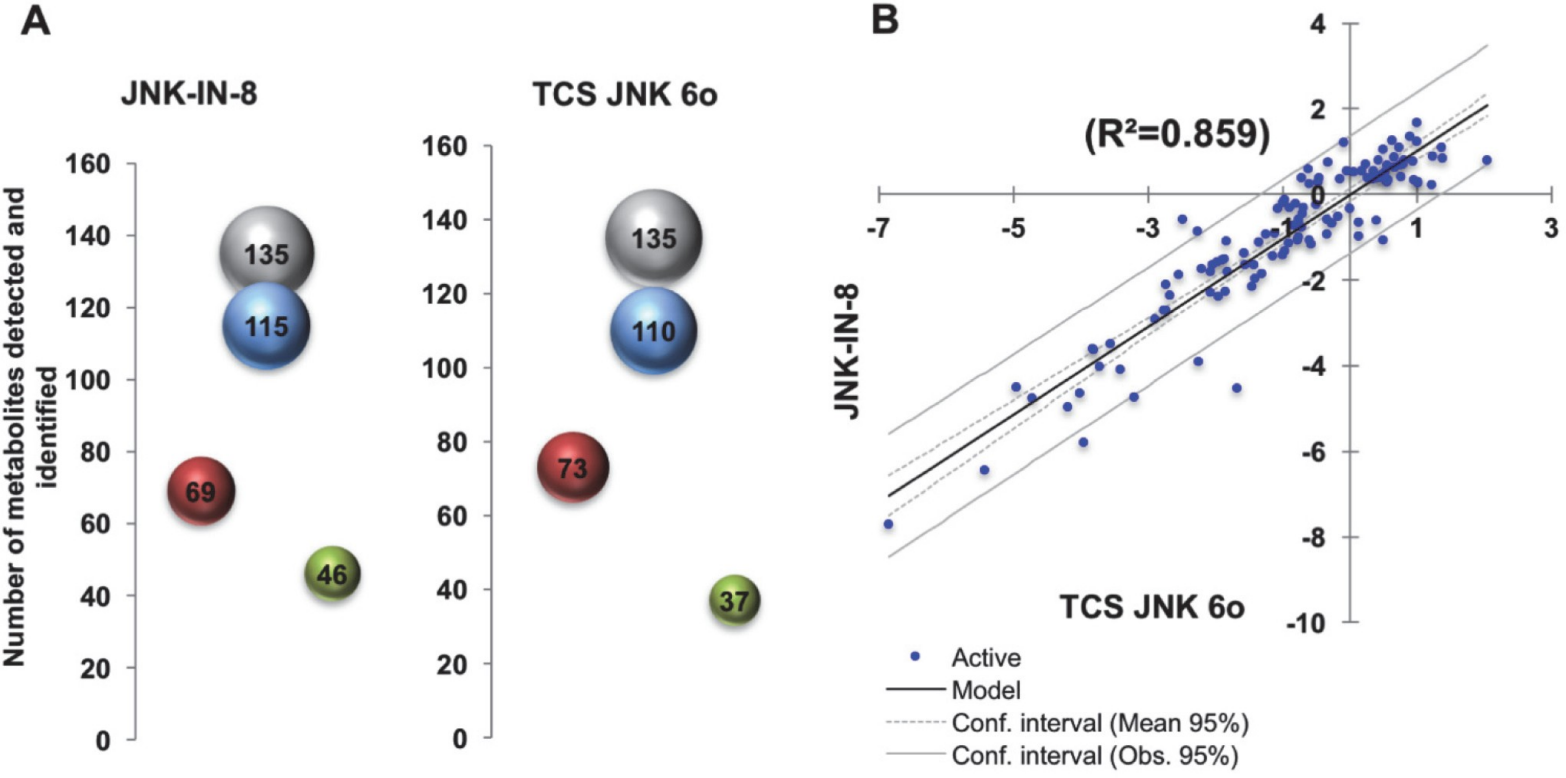
Midgut metabolites identified in *A*. *stephensi* treated with JNK SMIs. (**A**) 3-D bubble plot of the number of midgut metabolites detected in this study by GC-MS/MS. In grey, number of metabolites detected and identified; blue, number of metabolites with a different abundance than controls (LOG2 ratio >0.3 and <-0.3); in red, number of metabolites with higher concentrations than controls; green, number of metabolites with concentrations lower than controls. Bubble area is proportional to the number of metabolites (indicated at the center of each bubble). (**B**) Linear regression of the LOG2 ratios of metabolites associated with JNK-IN-8 and TCS JNK 6o treatments. A total of 81% of metabolites identified in both treatments showed an overlap (only 11.5% were unique to JNK-IN-8 and 7.3% to TCS JNK 6o). Both treatments resulted in similar metabolomes with relatively similar concentrations as judged by regression (r^2^ = 0.859; p < 0.001 Pearson’s; XLSAT).

For these studies, female *A*. *stephensi* were provided blood meals supplemented with 1 μM JNK-IN-8 or 1 μM TCS JNK 6o or an identical meal supplemented with an equivalent volume of diluent as a control. We selected 24 h post-feeding based on effects on midgut gene expression (**Fig 12**) to dissect and prepare midgut samples for analysis. Provision of JNK SMIs increased lactate levels (JNK-IN-8) and pyruvate levels (TCS JNK 6o) in the *A*. *stephensi* midgut relative to control (**S6 Fig**), whereas our previous studies showed that inhibition of p38 MAPK with the SMI BIRB796 increased lactate and decreased pyruvate [12], separating the effects of JNK and p38 MAPK on *A*. *stephensi* midgut intermediary metabolism. With GC-MS/MS of midguts from control mosquitoes and those treated with JNK SMIs, we detected and identified 135 metabolites (**Fig 16**). A large majority of compounds showed differential abundance in JNK SMI-treated versus control mosquitoes (85% for JNK-IN-8 and 81.5% for TCS JNK 6o; **Fig 16A**). Similar numbers of metabolites showed higher and lower abundances relative to controls for both JNK SMIs (60% and 66.4% higher in red, 40% and 33.6% lower in green for JNK-IN-8 and TCS JNK 6o, respectively, **Fig 16A**). Linear regression of the relative concentrations of metabolites for both inhibitors (**Fig 16B**) indicated that treatment with these two JNK SMIs resulted in analogous changes in metabolites and perhaps analogous pathways.

**Fig 17:**
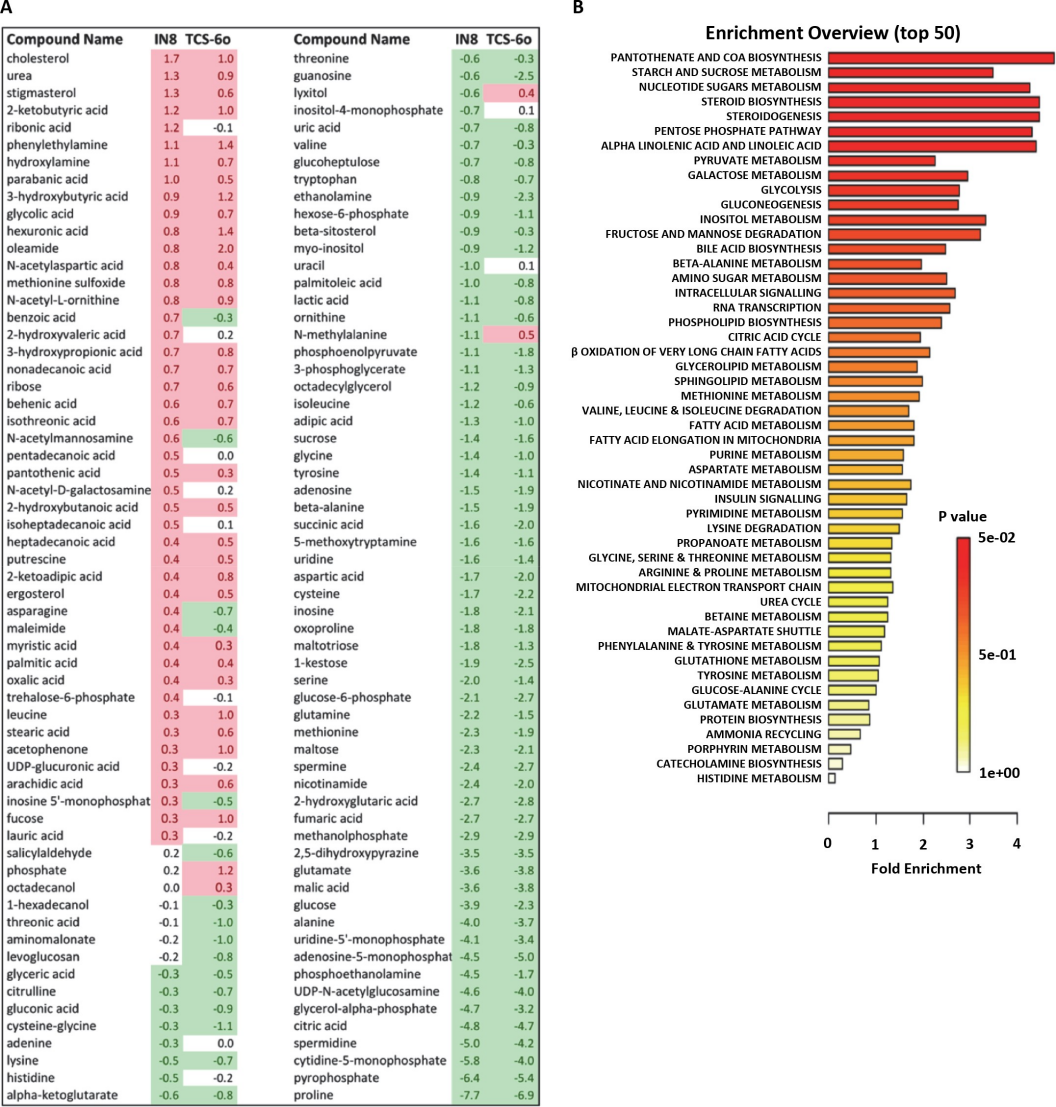
Identification of midgut metabolites in JNK SMI-treated *A*. *stephensi* with fold concentrations and metabolite set enrichment analysis. (A) Midgut metabolites identified by GC-MS/MS. Controls (n = 3) and treated samples (n = 3 per treatment) were processed to identify midgut metabolites. The relative concentrations of each metabolite were divided by the average of control levels and the resulting ratio was expressed as the LOG2. Cut-off for over-abundance was considered ≥ 0.3 whereas concentrations below that of controls were taken as ≤-0.3. (**B**) Summary plot for quantitative enrichment analysis (QEA). The QEA was performed using a generalized linear model to estimate a Q-statistic for each metabolite set, which describes the correlation between compound concentration and treatment. The right panel summarizes the average Q statistics for each metabolite in the input set.

Metabolites with different abundances in JNK SMI-treated mosquitoes versus controls (**Fig 17A**) along with their relative concentrations were analyzed using a Metabolite Set Enrichment Analysis (MSEA). Relative concentrations of metabolites were entered for quantitative enrichment analysis (QEA) using the metabolic pathway-associated metabolite set library and the GlobalTest package [51]. The QEA was performed using a generalized linear model to estimate a Q-statistic for each metabolite set, which described the correlation between compound concentration and treatment. The results are summarized as the average Q statistics for each metabolite in the input set (**Fig 17B**). Pathways significantly enriched were the pantothenate and coenzyme A (CoA) pathways, sucrose and nucleotide sugar metabolism, steroid synthesis, pentose phosphate shunt, fatty acid metabolism, pyruvate and galactose metabolism, and glycolysis and pyruvate metabolism (**Fig 17B**). The significant decrease in the ratio of active-to-total midgut pyruvate dehydrogenase complex (PDHC) suggested that JNK SMIs were inhibiting PDHC activity (**S6 Fig**). This was consistent with over-representation of the pantothenate pathway via metabolite-dependent inhibition of PDHC derived from increased acetyl CoA from fatty acid beta-oxidation to generate ketone bodies (*vide infra*).

**Fig 18:**
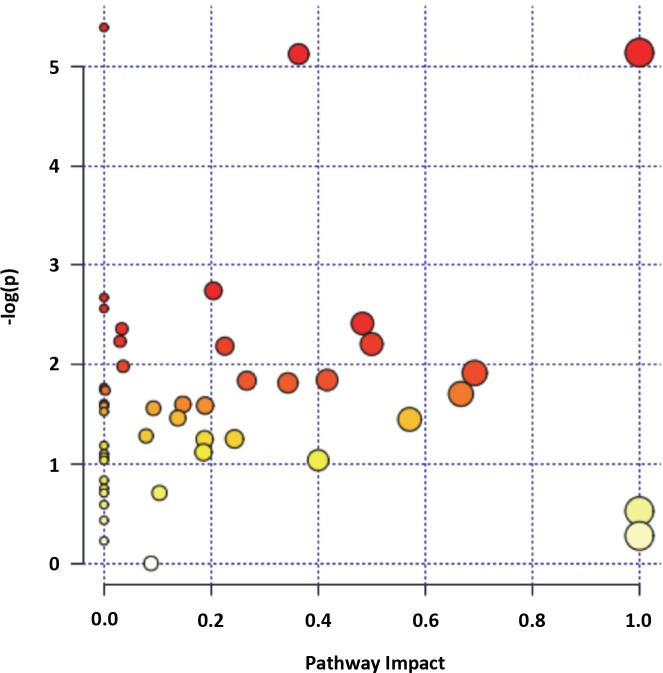
Pathway analysis of midgut metabolites identified in JNK SMI-treated *A*. *stephensi*. For comparison among different pathways, node importance values calculated from centrality measures were further normalized by the sum of the importance of the pathway. Therefore, the total/maximum importance of each pathway is 1. The importance measure of each metabolite node is the percentage of the total pathway importance, and the pathway impact value is the cumulative percentage from the matched metabolite nodes.

In parallel, we performed Pathway Analysis with normalized compound names using KEGG and PubChem databases for compound identification and the *D*. *melanogaster* pathway library. A detailed list of the pathways identified and their relative impacts are shown in **Table 4** and **Fig 18**, respectively. The most over-represented significant pathways (in decreasing order) were steroid biosynthesis, fatty acid metabolism, pentose phosphate shunt, inositol phosphate metabolism and ketogenesis. Thus, both analyses confirmed and extended the same conclusions: treatment with JNK SMIs was associated with over-representation of cholesterol, stigmasterol and ergosterol synthesis and over-representation of the CoA pathway, increased glucose flux through the pentose phosphate pathway (to sustain not only antioxidant defenses but also nucleic acid synthesis) and glucuronidation reactions at the expense of decreasing the flux through glycolysis. The abundances of all TCA cycle intermediates, including alpha-ketoglutarate, succinic acid, and citric acid were lower than control levels, suggesting reduced activity of the TCA cycle. Excess acetyl CoA (from glucose and ketogenic amino acids) not utilized in either the TCA cycle or cholesterol/sterol synthesis would be shunted to the formation of ketone bodies. A significantly lower concentration of most amino acids (16 of 17 detected) was observed likely because of proteolysis followed by the occurrence of anaplerotic reactions given the lower glucose flux to the TCA. In this regard, treatment with JNK SMIs is associated with catabolism of substrates other than glucose, including proline and trehalose, critical fuels for intermediary metabolism in mosquitoes [52].

**Table 4:** List of the pathways identified and their relative impacts.

Pathway	Total Compounds	Hits	Raw p	LN p	Holm adjust	FDR	Impact
Steroid biosynthesis	4	1	0.005	5.39	0.21942	0.096	0.000
Linoleic acid metabolism	6	1	0.006	5.14	0.27666	0.096	1.000
Pentose and glucuronate interconversions	14	2	0.006	5.12	0.27666	0.096	0.364
Inositol phosphate metabolism	24	2	0.064	2.74	1	0.385	0.204
Synthesis and degradation of ketone bodies	5	1	0.069	2.68	1	0.385	0.000
Trp metabolism	23	1	0.077	2.57	1	0.385	0.000
beta‐Ala metabolism	13	6	0.089	2.42	1	0.385	0.483
Glycerophospholipid metabolism	27	2	0.094	2.36	1	0.385	0.033
Glutathione metabolism	26	6	0.107	2.24	1	0.385	0.030
Ascorbate and aldarate metabolism	6	2	0.110	2.21	1	0.385	0.500
Pyrimidine metabolism	41	6	0.112	2.19	1	0.385	0.226
Sphingolipid metabolism	18	2	0.137	1.98	1	0.385	0.036
Phe metabolism	10	3	0.147	1.92	1	0.385	0.692
Glyoxylate and dicarboxylate metabolism	16	4	0.158	1.85	1	0.385	0.417
Propanoate metabolism	18	6	0.159	1.84	1	0.385	0.267
Arg and Pro metabolism	37	9	0.162	1.82	1	0.385	0.344
Butanoate metabolism	21	2	0.170	1.77	1	0.385	0.000
Val, Leu and Ile degradation	35	2	0.175	1.75	1	0.385	0.000
Amino sugar and nucleotide sugar metabolism	34	6	0.175	1.74	1	0.385	0.003
Val, Leu and Ile biosynthesis	13	4	0.181	1.71	1	0.385	0.667
Fatty acid biosynthesis	38	2	0.200	1.61	1	0.385	0.000
Citrate cycle (TCA cycle)	20	5	0.201	1.60	1	0.385	0.147
Purine metabolism	64	8	0.204	1.59	1	0.385	0.189
Fatty acid elongation in mitochondria	27	1	0.205	1.59	1	0.385	0.000
Fatty acid metabolism	38	1	0.205	1.59	1	0.385	0.000
Cys and Met metabolism	25	3	0.209	1.56	1	0.385	0.092
Nicotinate and nicotinamide metabolism	9	1	0.217	1.53	1	0.385	0.000
Aminoacyl‐tRNA biosynthesis	67	13	0.231	1.47	1	0.388	0.138
Gly, Ser and Thr metabolism	25	5	0.234	1.45	1	0.388	0.571
Galactose metabolism	26	3	0.277	1.29	1	0.429	0.079
Starch and sucrose metabolism	17	6	0.285	1.26	1	0.429	0.244
Tyr metabolism	30	2	0.286	1.25	1	0.429	0.188
Pentose phosphate pathway	19	2	0.304	1.19	1	0.443	0.000
Ala, Asp and Glu metabolism	23	5	0.326	1.12	1	0.446	0.186
Pantothenate and CoA biosynthesis	12	4	0.330	1.11	1	0.446	0.000
Lys degradation	17	2	0.342	1.07	1	0.446	0.000
Methane metabolism	9	2	0.353	1.04	1	0.446	0.400
Cyanoamino acid metabolism	6	2	0.353	1.04	1	0.446	0.000
Gln and Glu metabolism	5	1	0.432	0.84	1	0.531	0.000
Nitrogen metabolism	7	2	0.468	0.76	1	0.560	0.000
Glycolysis or Gluconeogenesis	25	2	0.490	0.71	1	0.560	0.104
Pyruvate metabolism	24	2	0.490	0.71	1	0.560	0.000
Porphyrin and chlorophyll metabolism	23	1	0.551	0.60	1	0.615	0.000
Phe, Tyr, and Trp biosynthesis	4	2	0.588	0.53	1	0.642	1.000
Ubiquinone and other terpenoid‐quinone biosynthesis	3	1	0.646	0.44	1	0.689	0.000
His metabolism	7	1	0.754	0.28	1	0.787	1.000
Biotin metabolism	5	1	0.794	0.23	1	0.811	0.000
Glycerolipid metabolism	16	1	0.994	0.01	1	0.994	0.088

**Fig 19:**
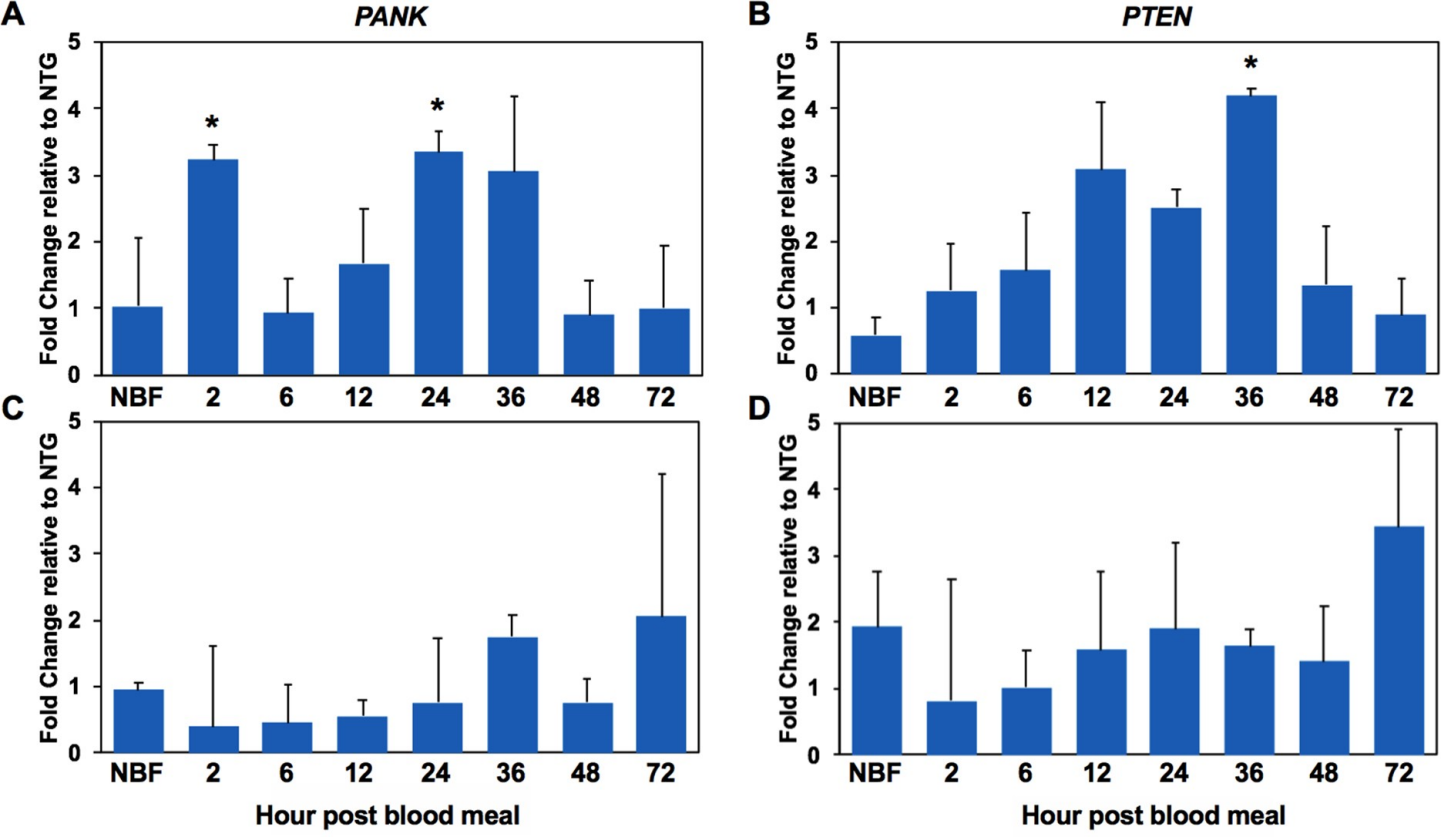
Expression of *PANK1* and *PTEN* genes in the M3 and M4 MKP4 transgenic *A*. *stephensi* lines. *PANK1* (**A**, **C**) and *PTEN* (**B**, **D**) expression levels were assessed by qPCR from pools of five mosquito midguts collected from 2–72 h during a reproductive cycle for the M3 (**A**, **B**) and M4 (**C**, **D**) MKP4 transgenic *A*. *stephensi* lines. All qPCR assays were performed in triplicate and normalized against ribosomal protein *S7*. The graphs show the fold change in *PANK* and *PTEN* expression levels between transgenic and non-transgenic sibling mosquitoes. Significant differences (p>0.05) are indicated with an asterisk. All qPCR experiments were replicated three times with distinct cohorts of mosquitoes.

The enrichment of pantothenate and CoA biosynthesis pathways suggested a mechanism for lifespan extension by inhibition of JNK signaling. Pantothenate kinase is the rate limiting enzyme for the conversion of pantothenate to CoA and previous work implicated upregulated *pantothenate kinase-1* (*pnk-1*) as a critical mediator of lifespan extension in long-lived *C*. *elegans daf-2* (insulin receptor ortholog) mutants [53, 54]. Further, knockdown of *pnk-1* using RNAi led to a threefold increase in the aging rate of *C*. *elegans* and dramatically shortened adult lifespan [53, 54]. Accordingly, we hypothesized that an increase in midgut pantothenate kinase would activate FOXO and inhibit IIS, which we have previously associated with lifespan extension and *P*. *falciparum* resistance in PTEN-overexpressing *A*. *stephensi* [14]. To assess this possibility in the context of a signaling manipulation that extended *A*. *stephensi* lifespan (**Fig 8**), we examined midgut mRNA expression of *A*. *stephensi pantothenate kinase* (*PANK)* and *PTEN* in the M3 and M4 transgenic lines at 2–72 h post blood meal (**Fig 19**). We observed significant increases in *PANK* at 2 h and 24 h post-blood meal and *PTEN* at 36 h post blood meal in the M3 line (**Fig 19A and 19B**), an effect that was consistent with increased MKP4 expression in this line (**Figs 5 and S1**). In contrast, neither *PANK* nor *PTEN* were induced in the midgut of the M4 line (**Fig 19C and 19D**) consistent with reduced resistance to *P*. *falciparum* (**Fig 11**) and lack of consistent lifespan extension (**Fig 8**) observed in this line relative to the M3 line. In sum, these data provided a novel link between JNK inhibition and IIS in the regulation of these life history traits.

## Discussion

With complementary experimental approaches, we have demonstrated that moderate inhibition of JNK signaling in the *A*. *stephensi* midgut extends lifespan and enhances resistance to the human malaria parasite *P*. *falciparum*. Resistance was independent of effects on NF-κB-dependent innate immunity, adding to the list of similar observations in *A*. *stephensi* that highlight the importance of alternative signaling pathways and intermediary metabolism in mediating anti-parasite resistance [14, 20, 22]. The phenotypic effects of JNK inhibition may be due in part to inhibition of IIS in the *A*. *stephensi* midgut, which we have previously associated with lifespan extension and resistance to parasite infection and which also reaffirms our observations of IIS-MAPK signaling cross-talk in the *A*. *stephensi* midgut [20, 22].

Importantly, our data contrast with previous studies in *A*. *gambiae* reporting that RNAi-mediated silencing of JNK and associated genes enhanced susceptibility, rather than resistance, to *Plasmodium berghei* infection [27]. Here, the authors assumed but did not confirm that hemipterous (MAP2K7) activates JNK signaling, they examined only a single *A*. *gambiae* JNK isoform (JNK1) despite prior identification of two JNK isoforms in this mosquito species [24], and assumed but did not confirm that Puckered specifically dephosphorylates JNK1 in *A*. *gambiae*. In *A*. *stephensi* cells, MKP5 (Puckered) decreased inducible activation of ERK, JNK, and p38 MAPK signaling (**Fig 4**), indicating that confirmation of MKP target specificity is critical for subsequent bioassays. In a follow on study, the same laboratory added JNK-interacting protein (JIP), with a single ortholog (JIP1) in *A*. *gambiae*, to their assays. Here, the authors reported that silencing of *JIP1*, *JNK* (*JNK1*), *Fos*, *Jun*, and *Puckered* had no effect on the prevalence or intensity of infection of *A*. *gambiae* with *P*. *falciparum* NF54, again without confirmation of protein-protein signaling interactions [26]. Notably, our colleagues asserted that *P*. *falciparum* Pfs47 was responsible for suppression of JNK signaling in *A*. *gambiae*, although this conclusion was based solely on patterns of infection with wild type and *Pfs47* knockout parasites in lacZ and JNK signaling RNAi-silenced mosquitoes [26]. No confirmatory analyses of JNK signaling or altered protein phosphorylation in any tissue were presented in support of these studies, making interpretations of these findings, as with earlier studies, difficult. Subsequent inferences from analyses of laboratory *P*. *falciparum* strains that specific Pfs47 mutations mediate “lock-and-key” relationships with geographically sympatric *Anopheles* spp. hosts were not supported by analyses of Pfs47 sequences in field isolates of *P*. *falciparum* and patterns of infections of sympatric mosquitoes with these isolates [55].

From our metabolomic analyses of JNK SMI-treated *A*. *stephensi*, pathways that were most enriched in the midgut included the pantothenate and CoA synthesis pathways and fatty acid metabolism. Accompanying these enrichments, a reduced flux through the TCA cycle was evidenced by lower concentrations of all intermediates relative to controls. These changes are supported by increased lactate and alanine per glucose, higher levels of pantothenic acid, increased fatty acid beta-oxidation, and lower levels of TCA cycle intermediates, which are maintained by anaplerotic reactions (see levels of key amino acids). A similar “low glycolytic state” versus “increased fatty acid oxidation state” was reported by us as functionally associated with reduced *P*. *falciparum* infection in *A*. *stephensi* [22]. Furthermore, transcription of *A*. *stephensi PANK*, which regulates the first critical step in CoA synthesis, was significantly upregulated in the midgut epithelium of the M3 line of MKP4 transgenic mosquitoes. *PANK* gene expression is directly controlled by the IIS transcription factor FOXO [53, 54]. Active IIS phosphorylates FOXO, excluding it from the nucleus and preventing it from transcribing target genes such as *PANK*. Conversely, suppression of the IIS cascade, for example through enhanced PTEN activity, leads to increased FOXO-dependent transcriptional activity and, as demonstrated in numerous organisms [56, 57], including *A*. *stephensi* [14], an increase in lifespan.

While these metabolic shifts supported enhanced resistance and lifespan, they were also associated with significantly reduced fecundity, patterns consistent with PTEN-mediated extension of lifespan and enhanced resistance with reduced lifetime fecundity in *A*. *stephensi* [14]. However, JNK inhibition was not associated with altered or enhanced midgut barrier integrity–as observed in PTEN overexpressing mosquitoes [14]–that could be consistently associated with enhanced resistance to *P*. *falciparum*. Accordingly, inhibited IIS could not fully explain the phenotypes associated with repression of JNK signaling in *A*. *stephensi*. In this context, we reasoned that metabolic shifts in *A*. *stephensi* resulting from JNK inhibition might directly affect *P*. *falciparum* development in the mosquito host. Intriguingly, several recent studies strongly support the likelihood of such direct effects on *P*. *falciparum* development.

William Trager, credited as the first individual to successfully continuously culture *P*. *falciparum*, also determined that panthothenate, as the precursor for CoA biosynthesis, was required for blood stage growth of several malaria parasite species, including *P*. *falciparum* [58]. This requirement is met by uptake of exogenous pantothenate because *P*. *falciparum* cannot synthesize pantothenic acid *de novo* [59, 60]. Using *P*. *yoelii* as a model, Hart et al. [61] characterized a parasite pantothenate transporter PyPAT that, upon knockout, had no effect on asexual development and gametocyte formation, but that blocked ookinete development and the formation of oocysts and sporozoites in *A*. *stephensi*. More recently, Hart et al. characterized two putative pantothenate kinase genes, *PyPanK1* and *PyPanK2*, from *P*. *yoelii* [62]. Knockouts of either gene target completed normal asexual development and gametocyte formation in mouse erythrocytes, affirming previous observations that some malaria parasite species could utilize alternative host precursors for CoA synthesis. However, knockout parasites were severely deficient in ookinete development and completely unable to produce sporozoites in *A*. *stephensi*, indicating that both PanK1 and PanK2 are required for the development of ookinetes and oocysts. Hart et al. also characterized CoA pathway enzymes in *P*. *yoelii*, noting that *de novo* biosynthesis is essential for both blood and mosquito stages of parasite development [63]. Taken together, these studies demonstrate that malaria parasites must take up pantothenate from the host to complete their CoA synthesis and these processes are essential for sporogony in the mosquito host. In this light, the upregulation of panthothenate, *PANK* expression and CoA synthesis in the *A*. *stephensi* midgut epithelium in response to JNK inhibition would likely compete directly with extracellular sporogonic parasites for these essential resources, suggesting for the first time that shifts in intermediary metabolism of mosquito host tissues may directly and adversely affect essential parasite metabolism. With the observations that pantothenate and CoA synthesis are essential for malaria parasite growth and development in both the mammalian and mosquito hosts, there is compelling interest in the development of novel pantothenate analogs and CoA synthesis-targeted inhibitors [64] as antimalarial compounds. To this we can add that targeted, genetic manipulations of intermediary metabolism of the mosquito host to synergize with these antimalarial compounds could provide enhanced confidence that transmission blocking could be complete.

## Materials and methods

### Mosquito MKP discovery

*Drosophila* MKP protein sequences were used to query the annotated *A*. *gambiae* genome [65] to uncover orthologues using Basic Local Alignment Search Tool (BLASTp). As an additional search strategy, vertebrate MKP protein sequences were used to query the *A*. *gambiae* genome. From both searches, six *A*. *gambiae* genes encoding proteins with significant similarities to the *Drosophila* and vertebrate MKPs were identified and used to identify *A*. *stephensi* orthologues (http://vectorbase.org). The extended consensus signature motifs of the MKP catalytic domain sequences from *A*. *gambiae*, *A*. *stephensi*, *D*. *melanogaster*, *Homo sapiens*, and *Mus musculus* were aligned using the MUltiple Sequence Comparison by Log-Expectation (MUSCLE) method. A neighbor-joining tree was constructed from the alignment using MEGA 5.05 software with the JTT model and 2000 bootstrap replications.

### Reagents

JNK-IN-8 [39] and TCS JNK 6o [40] were obtained from Selleck Chemicals (Houston, TX) and Tocris Bioscience (Minneapolis, MN), respectively. Antibodies used in this study were anti-GAPDH (G9545; Sigma-Aldrich) (1:10,000), and anti-pJNK1/2 Thr183+Tyr185 (44682G; Thermo Fisher Scientific, Waltham, MA) (1:3,000). Anti-mouse IgG-peroxidase (A9044; Sigma-Aldrich, St. Louis, MO) (GAPDH 1:20,000 and pJNK1/2 1:10,000) was used as a secondary antibody for immunoblotting.

### Mosquito cell culture, mosquito rearing, and mosquito feeding

The immortalized, *A*. *stephensi* embryo-derived (ASE) cell line (a gift from H.-M. Müller, EMBL) was maintained in modified minimal essential medium (MEM; Gibco, Invitrogen, Carlsbad, CA) with 5% heat-inactivated fetal bovine serum at 28°C under 5% CO_2_. *A*. *stephensi* mosquitoes were reared and maintained at 27°C and 80% humidity under a 12-hour light/dark cycle. Adult mosquitoes were maintained on 10% sucrose solution-soaked cotton pads and mice were used as a blood source for colony maintenance. Eggs were placed in water and larvae were fed a mixture of liquid food containing 2% w/v powdered fish food (Sera Micron) and baker’s yeast in a 2:1 ratio, and Game Fish Chow pellet food (Purina). All mosquito rearing protocols were approved and in accord with regulatory guidelines and standards set by the Institutional Animal Care and Use Committee of the University of California, Davis. For *in vivo* studies, 3 day old female mosquitoes were kept on water for 24 h and then allowed to feed for 30 min on an artificial blood meal of uninfected human erythryocytes resuspended in phosphate-buffered saline (PBS) provided through a Hemotek Insect Feeding System (Discovery Workshops, Accrington, UK). Chemical treatments were added to the erythrocyte-PBS mixture immediately prior to feeding (typically 1–5μl per ml of erythrocytes-PBS) with identical meals supplemented with an equivalent volume of diluent added for controls.

### Protein samples and immunoblotting

To harvest cellular proteins from *A*. *stephensi* ASE cells, cells were pelleted, washed with cold PBS, pelleted again, and then lysed in cell lysis buffer (10 mM Tris-HCl pH 7.4, 1mM EDTA, 100mM NaCl, 1mM NaF, 1mM EGTA, 2mM Na_3_VO_4_, 20 mM Na_4_P_2_O_7_, 0.1% SDS, 1% Triton X-100, 0.5% sodium deoxycholate, 1 mM phenylmethylsulfonyl fluoride, 10% glycerol, 60 mg/mL aprotinin, 10 mg/ml leupeptin, 1 mg/ml pepstatin, 1 mg/ml calyculin A). Cellular debris was removed by centrifugation at 14,000 × g for 20 min at 4°C. The resulting supernatants were mixed with Laemmli sample buffer (125 mM Tris-HCl pH 6.8, 10% glycerol, 10% SDS, 0.006% bromophenol blue, 130 mM dithiothreitol) and the proteins were denatured at 95°C for 3 min prior to electrophoresis.

Cellular proteins from mosquito midguts were processed as described previously [11]. Briefly, mosquito midguts were dissected into a protease inhibitor cocktail (Sigma) in ice-cold PBS and mixed to release blood, if any, by pipetting up and down. The midguts were washed in a filter column fitted with a fine mesh with the same PBS mixture until all the blood was removed. A final volume of the PBS mixture was added to loosen the midgut tissue from the filter and the tissue was transferred to a fresh tube and prepared for electrophoresis as described for cell culture lysates above.

Proteins were separated on 10% SDS-PAGE polyacrylamide gels at 135 V for 1 h, 40 min and then transferred to nitrocellulose membranes (BioRad Laboratories) for 1 h, 15 min at 7 V. Membranes were blocked in nonfat dry milk (5% w/v) in 1X Tris-buffered saline (TBS; pH 7.0) containing 0.1% Tween (TBS-T) for 1 h at room temperature, and then reacted overnight in primary antibody at 4°C. The membrane was washed 3 times, 5 min each with 1X TBS-T followed by incubation with appropriate secondary antibody at 4°C overnight. The membrane was washed again three times, 5 min each with 1X TBS-T and then incubated in SuperSignal West Dura Extended Duration Substrate (Pierce). The MultiDoc-It Imaging System and VisionWorks LS Image Acquisition software (UVP) were used to acquire membrane images. Densitometry analyses of antibody-bound proteins were performed using ImageJ software [66, 67]. Levels of phosphorylated JNK1 and JNK3 in each treatment were normalized to GAPDH levels to control for protein loading. For *in vitro* assays, pJNK1 and pJNK3 levels in treated cells were normalized to the same in diluent-treated control cells, whereas midgut pJNK1 and pJNK3 levels in treated or transgenic mosquitoes were normalized to GAPDH levels in the same samples, then to levels in control or non-transgenic mosquitoes to derive fold changes in phosphorylation.

### Plasmids, transfection, and luciferase reporter assays

Expression plasmids containing the complete coding sequences of *A*. *gambiae MKP3* [Genbank: XM_320303], *MKP4* [XM_320933], and *MKP5* [XM_001688405] followed by a V5 tag in the pDream2.1/MCS vector (Genscript, Piscataway, NJ) were transfected into ASE cells using Effectene reagent (Qiagen, Germantown, MD) according to the manufacturer’s protocol. In brief, 1×10^6^ cells in 1.6 ml medium were plated in 6-well tissue culture plates overnight at 28°C. At 24 h after plating, cells were transfected with 1 μg of plasmid DNA and incubated at 28°C.

*Cecropin*, *Defensin*, and *Gambicin* promoter-reporter plasmids were transfected into ASE cells as described [68, 69]. Briefly, 0.5×10^6^ cells in 0.25 ml medium were plated in 48-well tissue culture plates overnight at 28°C. At 24 h post-transfection, cells were treated with 1 μM JNK-IN-8 or TCS JNK 6o or an equivalent volume of diluent as a control for 1 h, and then challenged with 100 μg/ml of LPS (*Escherichia coli* serotype O26:B6; Sigma-Aldrich, St. Louis, MO). Luciferase activity was measured 24 h after immune challenge with the Dual-Glo system (Promega, Madison, WI).

### Development of CP-AsteMKP4-HA transgenic *A*. *stephensi*

Transgenic *A*. *stephensi* lines overexpressing MKP4 under the control of the midgut-specific carboxypeptidase (CP) promoter were created with an HA epitope at the carboxyl terminus (AsteMKP4-HA) to facilitate protein identification. The construct was inserted into the pBac[3XP3-EGFPafm] plasmid vector using *Asc*I. The construct was sent to the University of Maryland Biotechnology Institute, Insect Transformation Facility (UMBI-ITF) for transformation of *A*. *stephensi*. A total of 10 EGFP-positive *A*. *stephensi* lines were generated. We chose two representative lines, M3 and M4, based on strong (M3) and moderate (M4) midgut-specific transcript and protein expression patterns. Transgene insertion sites in the mosquito genome were identified by inverse PCR following the protocol of Buchholz et al. [70]. Transgenic mosquitoes were maintained as hemizygous lines by outcrossing the mosquitoes in each generation to colony non-transgenic *A*. *stephensi* in order to maximize genetic diversity.

### Lifespan studies and reproductive assays

For experiments using JNK SMIs, female *A*. *stephensi* were maintained in cartons and provided artificial blood meals once per week for the duration of their lifespans. Each replicate was initiated with 300 mosquitoes and blood meals were supplemented with JNK-IN-8, TCS JNK 6o, or an equivalent volume of diluent added to the blood meal as a control. Dead mosquitoes were counted three times per week and oviposition cups provided after each blood feeding. These experiments were replicated with separate cohorts of mosquitoes.

Mosquitoes hemizygous for the CP-AsteMKP4-HA construct were mated with non-transgenic *A*. *stephensi* to generate 50% transgenic and 50% non-transgenic sibling mosquitoes. The resulting larvae were reared together under identical conditions and separated based on EGFP eye fluorescence of pupae under a fluorescent stereomicroscope. Mosquitoes were provided uninfected blood meals three times per week for the duration of adult life in addition to 10% sucrose *ad libitum*. Daily mortality for each treatment was recorded and dead mosquitoes were removed until all mosquitoes had perished. These experiments were replicated a minimum of three times using separate cohorts.

Because of cost, the effects of JNK SMIs on fecundity were examined only for the first gonotrophic cycle. To this end, 3–5 day-old female *A*. *stephensi* were provided an artificial blood meal supplemented with JNK-IN-8, TCS JNK 6o, or an equivalent volume of diluent added to the blood meal as a control. Mosquitoes that did not feed or only partially fed were discarded. At 48 h after feeding, females were individually housed in modified 50 ml conical tubes and provided water for oviposition. At 72–96 h after blood feeding, eggs were collected from each conical tube, washed onto filter paper and photographed. Eggs were then counted using ImageJ software [66, 67].

To study the effects of transgene expression on lifetime fecundity, hemizygous CP-AsteMKP4-HA and non-transgenic controls were maintained under identical conditions. Briefly, groups of 150 transgenic and non-transgenic *A*. *stephensi* females were mated with 50 non-transgenic males. Each cage of 150 female mosquitoes was blood fed daily throughout their lifespans to ensure equal blood feeding success. Damp filter paper for oviposition was provided at 48 h after the first blood meal and replaced every 24 h if eggs were present. Eggs from transgenic and non-transgenic *A*. *stephensi* cages were counted using ImageJ software. This experiment was replicated a minimum of three times with separate cohorts.

### *P*. *falciparum* culture, mosquito infection, and parasite growth assays

For mosquito infection studies, the NF54 strain of *P*. *falciparum* was initiated at 1% parasitemia in 10% heat-inactivated human serum and 6% washed red blood cells in RPMI 1640 with HEPES (Gibco, Invitrogen) and hypoxanthine. At days 15–17, stage V gametocytes were evident and exflagellation was evaluated at 200× magnification with phase-contrast or modified brightfield microscopy the day before and day of feeding by observation of blood smears and before addition of fresh media. Female *A*. *stephensi* were provided water only (no sucrose) for 24 h and then fed a meal of *P*. *falciparum*-infected erythrocytes. Mosquitoes (n = 150 per group) were given 30 min to feed, after which non-fed and partially fed mosquitoes were removed, and those that were fully engorged were maintained on cotton pads soaked in 10% sucrose until day 10 post-infection. On day 10, midguts were dissected and stained with 0.5% mercurochrome for visualization of *P*. *falciparum* oocysts by microscopy. These experiments were replicated using four separate *P*. *falciparum* cultures and separate cohorts of *A*. *stephensi*.

For *P*. *falciparum* growth assays, NF54 cultures were synchronized with sorbitol and aliquots were plated in complete RPMI 1640 medium with HEPES, hypoxanthine, and 10% heat inactivated human serum. Parasites were treated with 0.1, 1, 10 μM JNK-IN-8 or TCS JNK 6o or an equivalent volume of diluent as a control and incubated at 37°C for 48 or 96 h before culture media were replaced with 10% formalin in RPMI 1640. RBCs were stained with 10 μg/mL propidium iodide (Sigma-Aldrich) at room temperature for 1 h and infected cells counted with FACSCalibur flow cytometer (BD Biosciences, San Jose, CA). These assays were replicated four times with separate parasite culture passages.

### Real-time quantitative PCR of mRNA transcripts

Transgene expression patterns were examined in the midgut epithelium and carcass (body minus midgut) in non-transgenic and transgenic female *A*. *stephensi* (3–5 day old) before and 24 h post blood feeding. Additionally, transgene transcripts were amplified in midgut tissue at 2, 6, 12, 24, 36, 48, and 72 h post-blood feeding in transgenic mosquitoes. In both experiments, total RNAs were isolated from the midguts and carcasses (RNeasy; Qiagen) and converted into cDNA (High-Capacity cDNA Reverse Transcription Kit; Thermo) per the manufacturer’s instructions Primers specific to *MKP4* (MKP4-HA-For: TCCGAAGTGTACCGTGAAGA; MKP4-HA-Rev: AGCGTAATCTGGCACATCGT) and *actin* (Actin-For: AGCGTGGTATCCTGACGCTGAAAT; Actin-Rev AACCTTCGTAGATCGGCACGGTAT) were used.

To investigate anti-parasite gene mRNA expression patterns, transgenic and non-transgenic female *A*. *stephensi* (3–5 day old) were fed identical *P*. *falciparum*-infected blood meals that were untreated or treated with JNK SMIs or diluent and, at 3 h and 24 h post-blood feeding, 15 midguts were pooled and dissected into cold PBS. Total RNA was isolated and reverse transcribed into cDNA as above. Primers for *A*. *stephensi APL1*, *Defensin*, *LRIM1*, *LRRD7*, *NOS*, and *TEP1* were as previously described [14]. To investigate IIS regulation by MKP4 overexpression, we examined the mRNA expression of *PANK* and *PTEN* at the same time points described above for transgene expression using primer sets for *PANK* (PANK-For: GCCAACCGTACCCGTTTAT; PANK-Rev: CGGAGATGCGCTTGTAGTT) and *PTEN* (PTEN-For: GCTTCACCAGTAATCGCAGTA; PTEN-Rev: GGTGGCCTAGCGTCTAAATTAT).

Anti-parasite gene mRNA expression assays were performed using Maxima SYBR Green/ROX qPCR Master Mix (Fermentas, Waltham, MA) and an ABI Prism 7300 Sequence Detection System (Applied Biosystems, Foster City, CA). The amplification conditions were as follows: initial denaturation at 95°C for 2 min, followed by 40 cycles of denaturation for 15 s at 95°C and annealing/extension at 60°C for 30 sec. Amplification results were analyzed by normalizing the data to the mosquito housekeeping gene ribosomal protein *S7*. For *PANK* and *PTEN* qPCR expression analysis, Maxima SYBR Green/ROX qPCR Master Mix (Fermentas, Waltham, MA) was used with an Eppendorf RealPlex2 Mastercycler (Eppendorf, Hauppauge, NY). The amplification conditions were as follows: initial denaturation at 95°C for 3 min, followed by 40 cycles of denaturation for 30 s at 95°C and annealing/extension at 60°C for 30 sec. Amplification results were analyzed by normalizing the data to the mosquito housekeeping gene ribosomal protein *S7*.

### Functional assays of midgut permeability in JNK SMI-fed and in CP-AsteMKP4-HA *A*. *stephensi*

Three-day old female *A*. *stephensi* were maintained on water for 48 h and then allowed to feed for 30 min on artificial blood meals (human erythrocytes in PBS) with 2×10^6^ magnetic fluorescent particles/ml (2.0–2.4 μm, Fluorescent Yellow Carboxyl Magnetic Particles FCM-2052-2; Spherotech, Lake Forest, IL). Blood meals with fluorescent particles were supplemented with equivalent volumes of 1 μM JNK-IN-8, 1 μM TCS JNK 6o, or diluent (DMSO) as a control for SMI-treated mosquitoes. Non-fed mosquitoes were immediately removed after feeding. At 72 h post-feeding, samples of five pooled whole mosquitoes or five pooled dissected midguts were placed in sterile water, pulse-sonicated, and passed through a 35 μm filter to remove tissue debris. Samples were then transferred into 1.5 ml microcentrifuge tubes. Fluorescent particles were collected using the MagnaRack Magnetic Separation Rack (Thermo Fisher Scientific) and washed twice with sterile water, then collected into a final volume of 5–10 μl sterile water. AbsorbMax Black Sealing Film (Sigma) was cut to size and applied to the bottom of a diagnostic slide with white Teflon printed circles. The magnetic fluorescent particles were pipetted onto the circles and allowed to dry. Images were acquired with a stereoscope with fluorescence (Olympus) and processed and analyzed using Sketchbook Pro (Autodesk, San Rafael, CA) and ImageJ software. To estimate the number of particles that passed through the midgut epithelial tissue and into the body cavity, the numbers of particles in the five midguts were subtracted from each analyzed sample of five whole mosquitoes, removing the contribution of beads remaining in the midgut of whole body samples. The experiment was replicated with three separate cohorts of mosquitoes.

### Metabolomics analyses of JNK SMI-treated *A*. *stephensi*

Mosquitoes were provided artificial blood meals supplemented with 1 μM JNK-IN-8, 1 μM TCS JNK 6o, or with an equivalent volume of diluent as a control. At 24 h after feeding, midguts were dissected from 100 mosquitoes in each treatment and control, collected into HEPES buffer with protease inhibitor cocktail in ice-cold PBS, and pulse sonicated. Samples were extracted following published protocols [71]. Thirty μl aliquots were extracted by 1 ml of degassed acetonitrile:isopropanol:water (3:3:2, v/v/v) at -20°C, centrifuged, decanted, and evaporated to complete dryness prior to a clean-up step with acetonitrile in water (1:1). The cleaned extract was aliquoted into two equal portions and the supernatant was dried down again. Internal standards (C08-C30 fatty acid methyl esters) were added and the sample was derivatized by methoxyamine hydrochloride in pyridine and subsequently by N-methyl-N-trimethylsilyltrifluoroacetamide for trimethylsilylation of acidic protons. Data were acquired using the following chromatographic parameters [72]: Column: Restek corporation rtx5Sil-MS (30 m length x 0.25 mm internal diameter with 0.25 μm film of 95% dimethyl- and 5% diphenyl-polysiloxane); Mobile phase: helium; Column temperature: 50–330°C; Flow-rate: 1 ml min^-1;^ Injection volume: 0.5 μl; Injection: 25 splitless time into a multi-baffled glass liner; Injection temperature: 50°C ramped to 250°C by 12°C s^-1^; Gradient: 50°C for 1 min, then ramped at 20°C min^-1^ to 330°C, held constant for 5 min. Mass spectrometry parameters were as follows: a Leco Pegasus IV mass spectrometer was used with unit mass resolution at 17 spectra s^-1^ from 80–500 Da at -70 eV ionization energy and 1800 V detector voltage with a 230°C transfer line and a 250°C ion source. Data processing: Raw data files were preprocessed directly after data acquisition and stored as ChromaTOF-specific *.peg files, as generic *.txt result files and additionally as generic ANDI MS *.cdf files. ChromaTOF vs. 2.32 was used for data preprocessing without smoothing, 3 sec peak width, baseline subtraction just above the noise level, and automatic mass spectral deconvolution and peak detection at signal/noise levels of 5:1 throughout the chromatogram. Apex masses were reported for use in the BinBase algorithm. Resulting *.txt files were exported to a data server with absolute spectra intensities and further processed by a filtering algorithm implemented in the metabolomics BinBase database. The BinBase algorithm used the settings: validity of chromatogram (<10 peaks with intensity greater than 10^7 counts per sec), unbiased retention index marker detection (MS similarity >800, validity of intensity range for high m/z marker ions), retention index calculation by 5th order polynomial regression. Spectra were cut to 5% base peak abundance and matched to database entries from most to least abundant spectra using the following matching filters: retention index window ±2,000 units (equivalent to about ±2 sec retention time), validation of unique ions and apex masses (unique ion must be included in apexing masses and present at >3% of base peak abundance), mass spectrum similarity fit to criteria dependent on peak purity and signal/noise ratios and a final isomer filter. Failed spectra were automatically entered as new database entries if s/n >25, purity <1.0 and presence in the biological study design class was >80%. All thresholds reflect settings for ChromaTOF vs. 2.32. Quantification was reported as peak height using the unique ion as default, unless a different quantification ion was manually set in the BinBase administration software BinView. A quantification report table was produced for all database entries that were positively detected in more than 10% of the samples of a study design class (as defined in the miniX database) for unidentified metabolites. A subsequent post-processing module was employed to automatically replace missing values from the *.cdf files. Replaced values were labeled as “low confidence” by color coding, and for each metabolite, the number of high-confidence peak detections was recorded as well as the ratio of the average height of replaced values to high-confidence peak detections. These ratios and numbers were used for manual curation of automatic report data sets to data sets released for submission. Metabolites were identified by matching the ion chromatographic retention index, accurate mass, and mass spectral fragmentation signatures with reference library entries created from authentic standard metabolites under the identical analytical procedure as the experimental samples.

### Statistical analyses

Relative levels of phosphorylated JNK1 and JNK3 normalized to GAPDH were analyzed using one-way ANOVA. Differences in JNK1 and JNK3 phosphorylation levels between SMI-treated and untreated mosquitoes and between transgenic and non-transgenic *A*. *stephensi* were determined by Student’s t-test. Survival analyses for JNK SMI-treated mosquitoes and controls and for transgenic and non-transgenic *A*. *stephensi* were performed using the Gehan-Breslow-Wilcoxon method. Analysis of median lifespans from independent replicates of JNK SMI-treated *A*. *stephensi* and MKP4 transgenic mosquitoes relative to controls was performed using one-way ANOVA. The proportions of females that laid eggs in the first gonotrophic cycle (JNK SMI-treated versus controls) were compared using Chi-square test, while egg clutch sizes per female in these studies were analyzed using Mann-Whitney test. Lifetime fecundity per female or for cages or populations of M3 and M4 lines of MKP4 transgenic *A*. *stephensi* relative to controls were calculated using Student’s t-test. Infection data were initially analyzed by ANOVA to determine whether mean oocysts (for mosquitoes with at least one midgut oocyst) differed among *A*. *stephensi* cohorts. No significant differences were found, so infection intensity data were analyzed using Kruskal-Wallis and Dunn’s post-test. Infection prevalences were analyzed by Chi-square test. Relative changes in *P*. *falciparum* growth were normalized to controls set at one and analyzed by Student’s t-test. mRNA expression data were analyzed by Student’s t-test to determine significance of changes relative to GAPDH (for age-associated patterns) or by one-way ANOVA for JNK SMI treatments and controls and for transgenic and non-transgenic *A*. *stephensi*. Luciferase activity levels of treatments and controls were analyzed by Student’s t-test. Fluorescent particle data for JNK SMI-treated *A*. *stephensi* and for transgenic and non-transgenic mosquitoes were analyzed by one-way ANOVA. All differences were considered statistically significant at α < 0.05.

## Supporting information

S1 Fig The M3 MKP4 transgenic *A. stephensi* expressed significantly more MKP4-HA protein than the M4 line. Immunoblots were performed on the midguts of M3 and M4 mosquitoes prior to blood feeding (0 h) and 24 hours after blood feeding (24 h). While M3 line mosquitoes had more MKP4-HA protein expressed both before and after blood feeding, the increased expression was only significant at 24 hours after blood feeding. Immunoblots were replicated five times with unique cohorts of mosquitoes. For all replicates, M3 and M4 samples were processed on a single immunoblot to allow for direct comparisons of transgene expression.
(TIFF)

S2 Fig Identification of the *MKP4* genomic insertion site in transgenic *A. stephensi*. Inverse PCR was used to identify the 5’ and 3’ sequences surrounding the MKP4 construct in the *A*. *stephensi* M3 line. A schematic of inverse PCR product sequence is shown. Transgenic genomic DNA was cut with *Mbol*, self-ligated and used as a PCR template with piggybac (pBac) specific primers. The amplified product (M3 370bp; M4 125bp) from the putative insertion site was flanked with known pBac sequence. Sequencing the resulting inverse PCR fragments demonstrated that the transgene inserted into a TTAA sequence, the preferred site of pBac transposition, and did not disrupt any known or predicted *A*. *stephensi* genes.
(TIFF)

S3 Fig The conserved ATP binding site in human and *A. stephensi* JNK isoforms. Human (Hs) JNK1, JNK2, and JNK3 and *A*. *stephensi* (As) JNK1 and JNK3 show significant overall conservation among the residues that compose the ATP binding site (black triangles). The cysteine residue with which JNK-IN-8 forms a covalent bond (open circle) is conserved in *A*. *stephensi* JNK1. Human JNK1 [Genbank: NP_001310231], human JNK2 [Genbank: NP_002743], human JNK3 [Genbank: AAH51731], *A*. *stephensi* JNK1 and JNK3 protein sequences (ASTE007551 and ASTE007552, respectively) were aligned using the MUSCLE method with default settings in Geneious (“Geneious version 5.5.8 created by Biomatters”).
(TIFF)

S4 Fig MKP4 midgut expression suppresses p38 phosphorylation following an infectious bloodmeal, but has no impact on ERK phosphorylation. Non-transgenic (NTG) and MKP4 transgenic *A*. *stephensi* (M3) were assayed for p38 (A) and ERK (B) phosphorylation prior to bloodfeeding (NBF) and after being provided with a *P*. *falciparum* infected bloodmeal (Pf). ERK and p38 phosphorylation levels were assessed using immunblot assays at 3 h after the infectious bloodmeal. GAPDH was used as a loading control and results were normalized to the NBF controls. Assays were replicated twice.
(TIF)

S5 Fig JNK SMIs at levels tested *in vivo* do not directly affect *P. falciparum* asexual growth *in vitro*. Synchronized, asexual stage *P*. *falciparum* parasites were treated with increasing concentrations of JNK SMIs and growth was evaluated at 48 and 96 h post treatment. Relative growth was normalized to parasites treated with diluent control (set at 1, dashed line). Pairwise comparisons of treatments and matched controls were analyzed by Student’s t-test, *P < 0.05. These assays were replicated four times with separate parasite culture passages.
(TIFF)

S6 Fig Levels of midgut lactate, pyruvate and the ratio of active-to-total pyruvate dehydrogenase complex activity in JNK SMI-treated and control *A. stephensi*. Samples were evaluated as described utilizing enzymatic assays [73]. Data were analyzed by ANOVA followed by Bonferroni’s post-hoc analysis.
(TIFF)
