# Supplementary figures and images for: Inhibition of JNK signaling in the Asian malaria vector Anopheles stephensi extends mosquito longevity and improves resistance to Plasmodium falciparum infection
Source: PLoS Pathog. 2018 Nov 29;14(11):e1007418. doi: 10.1371/journal.ppat.1007418 (PMC6264519; doi:10.1371/journal.ppat.1007418)

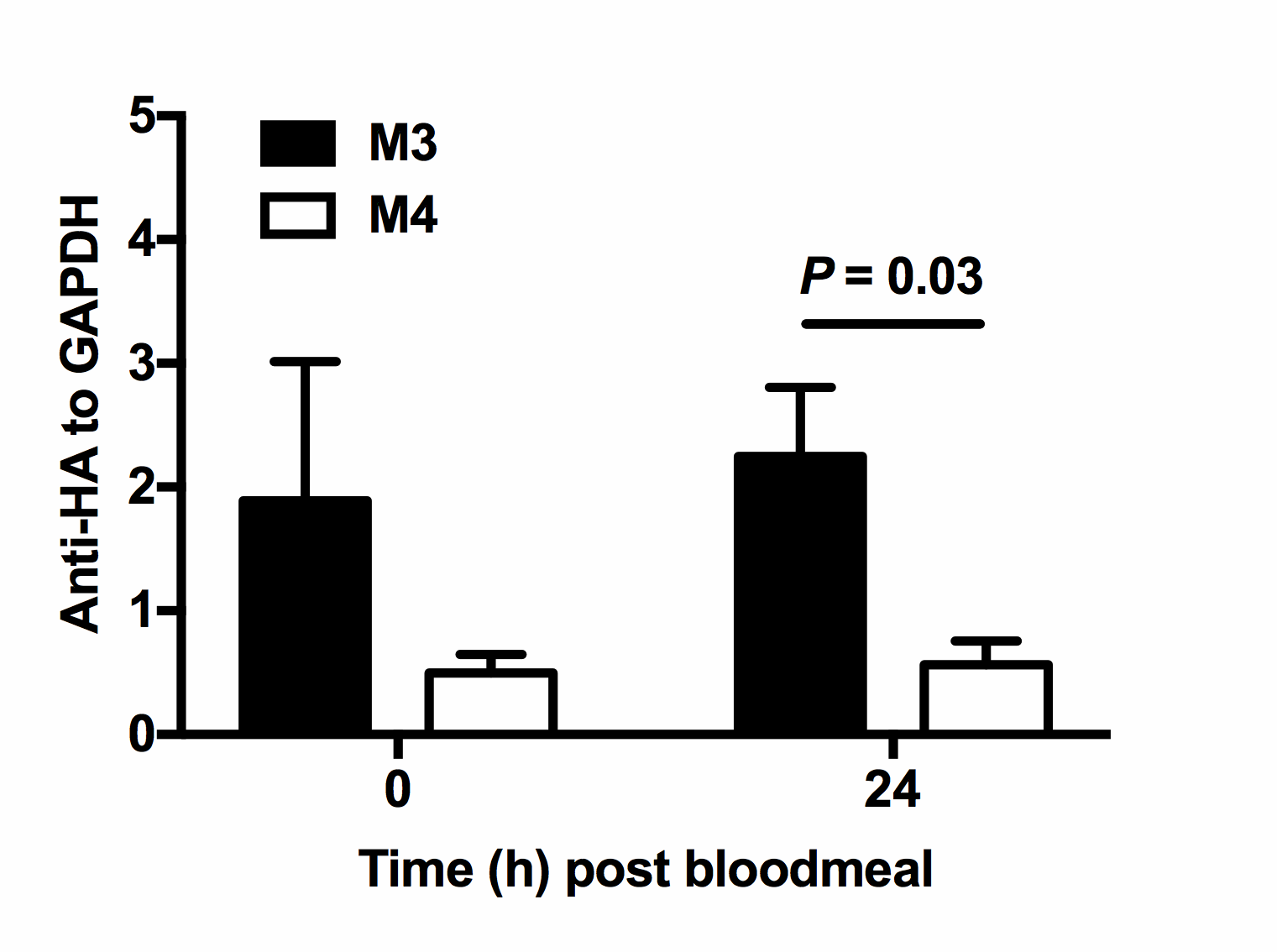

Supplement: S1 Fig — Immunoblots were performed on the midguts of M3 and M4 mosquitoes prior to blood feeding (0 h) and 24 hours after blood feeding (24 h). While M3 line mosquitoes had more MKP4-HA protein expressed both before and after blood feeding, the increased expression was only significant at 24 hours after blood feeding. Immunoblots were replicated five times with unique cohorts of mosquitoes. For all replicates, M3 and M4 samples were processed on a single immunoblot to allow for direct comparisons of transgene expression. (TIFF) [file ppat.1007418.s001.tiff]

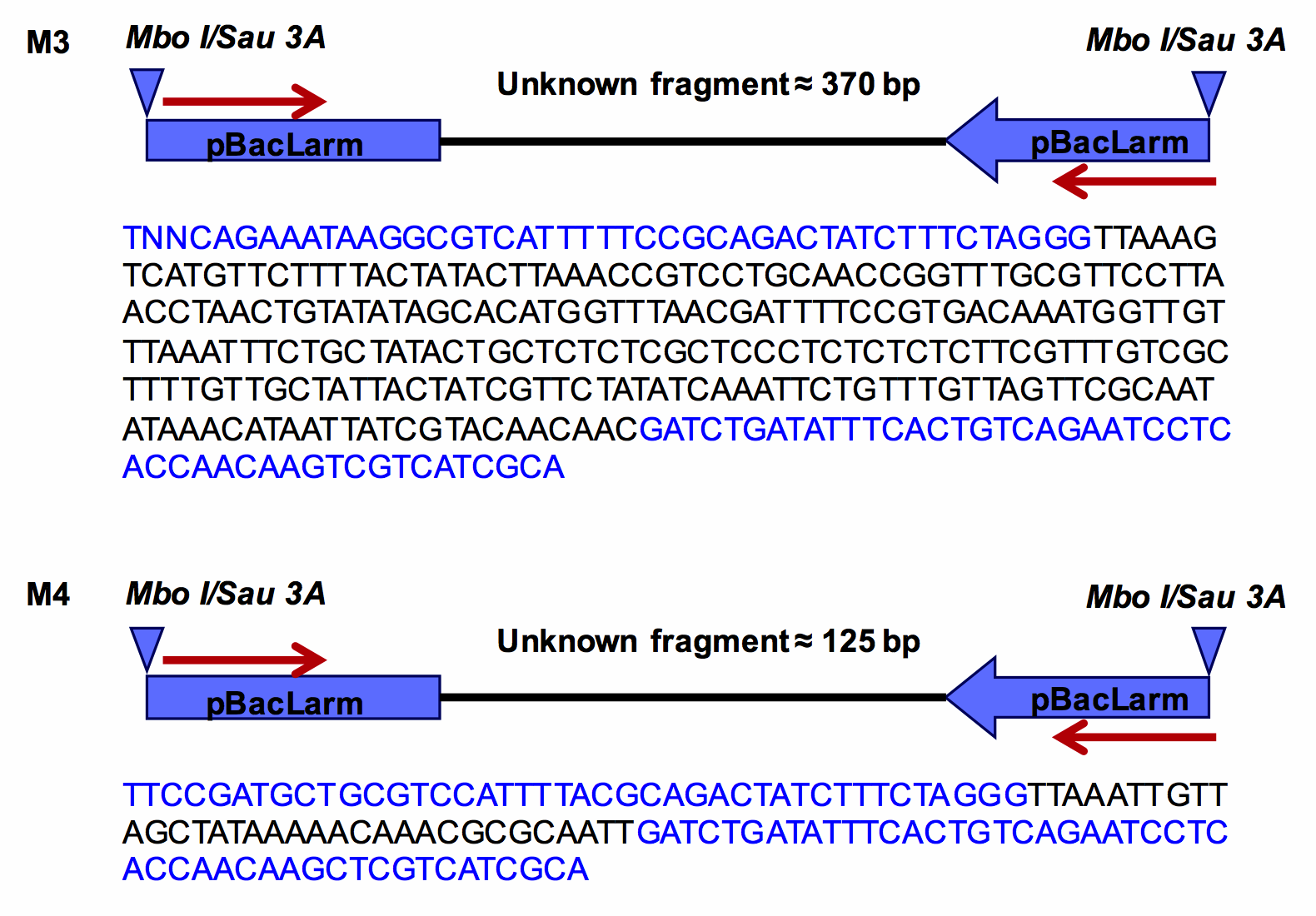

Supplement: S2 Fig — Inverse PCR was used to identify the 5’ and 3’ sequences surrounding the MKP4 construct in the A. stephensi M3 line. A schematic of inverse PCR product sequence is shown. Transgenic genomic DNA was cut with Mbol, self-ligated and used as a PCR template with piggybac (pBac) specific primers. The amplified product (M3 370bp; M4 125bp) from the putative insertion site was flanked with known pBac sequence. Sequencing the resulting inverse PCR fragments demonstrated that the transgene inserted into a TTAA sequence, the preferred site of pBac transposition, and did not disrupt any known or predicted A. stephensi genes. (TIFF) [file ppat.1007418.s002.tiff]

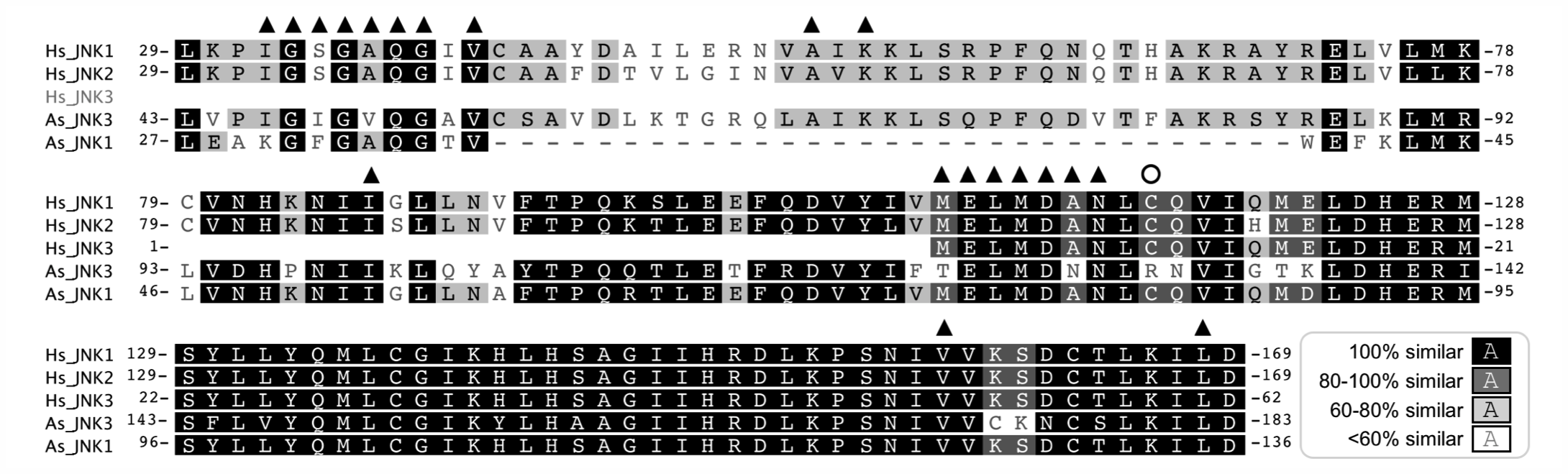

Supplement: S3 Fig — Human (Hs) JNK1, JNK2, and JNK3 and A. stephensi (As) JNK1 and JNK3 show significant overall conservation among the residues that compose the ATP binding site (black triangles). The cysteine residue with which JNK-IN-8 forms a covalent bond (open circle) is conserved in A. stephensi JNK1. Human JNK1 [Genbank: NP_001310231], human JNK2 [Genbank: NP_002743], human JNK3 [Genbank: AAH51731], A. stephensi JNK1 and JNK3 protein sequences (ASTE007551 and ASTE007552, respectively) were aligned using the MUSCLE method with default settings in Geneious (“Geneious version 5.5.8 created by Biomatters”). (TIFF) [file ppat.1007418.s003.tiff]

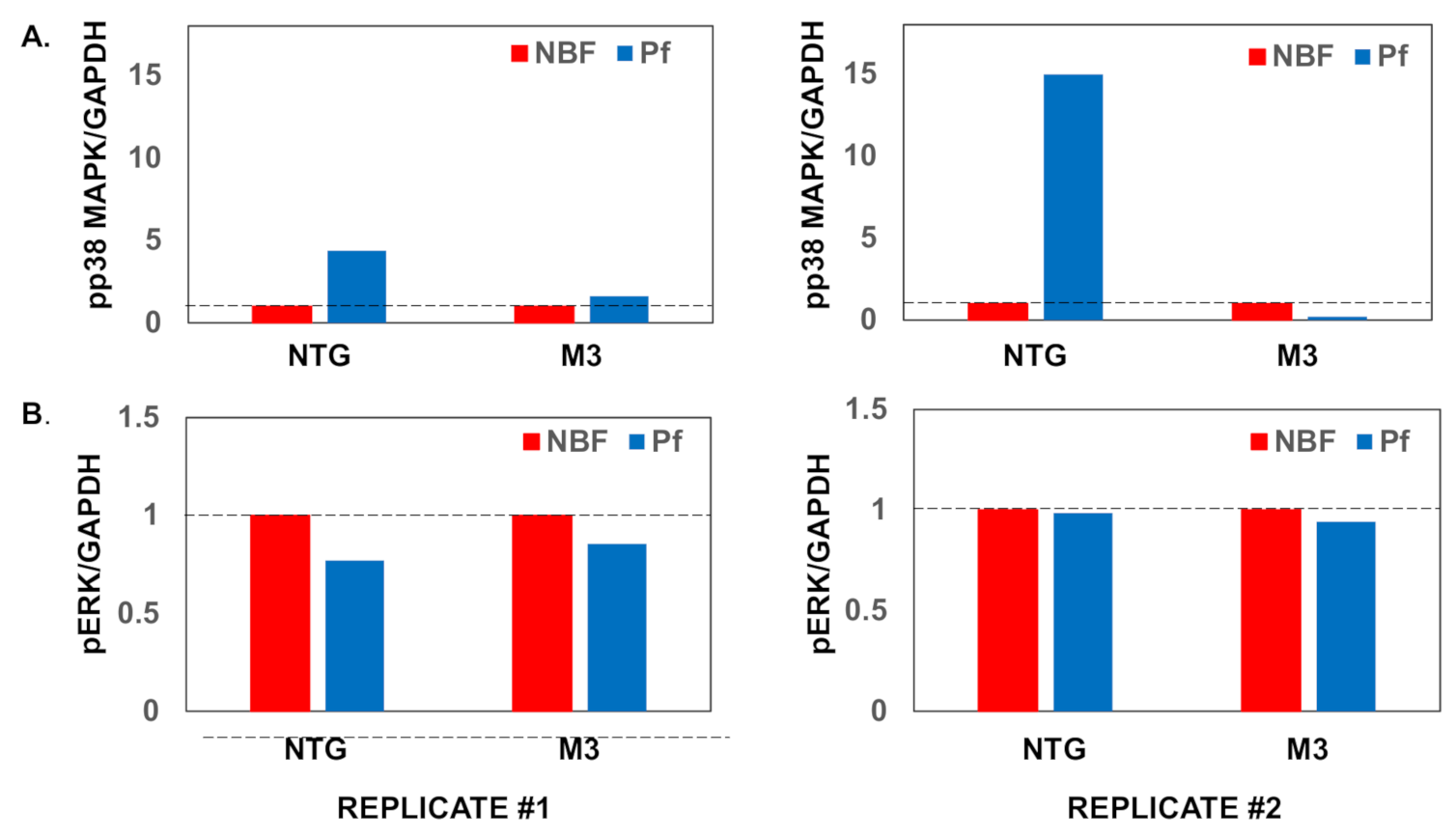

Supplement: S4 Fig — Non-transgenic (NTG) and MKP4 transgenic A. stephensi (M3) were assayed for p38 (A) and ERK (B) phosphorylation prior to bloodfeeding (NBF) and after being provided with a P. falciparum infected bloodmeal (Pf). ERK and p38 phosphorylation levels were assessed using immunblot assays at 3 h after the infectious bloodmeal. GAPDH was used as a loading control and results were normalized to the NBF controls. Assays were replicated twice. (TIF) [file ppat.1007418.s004.tif]

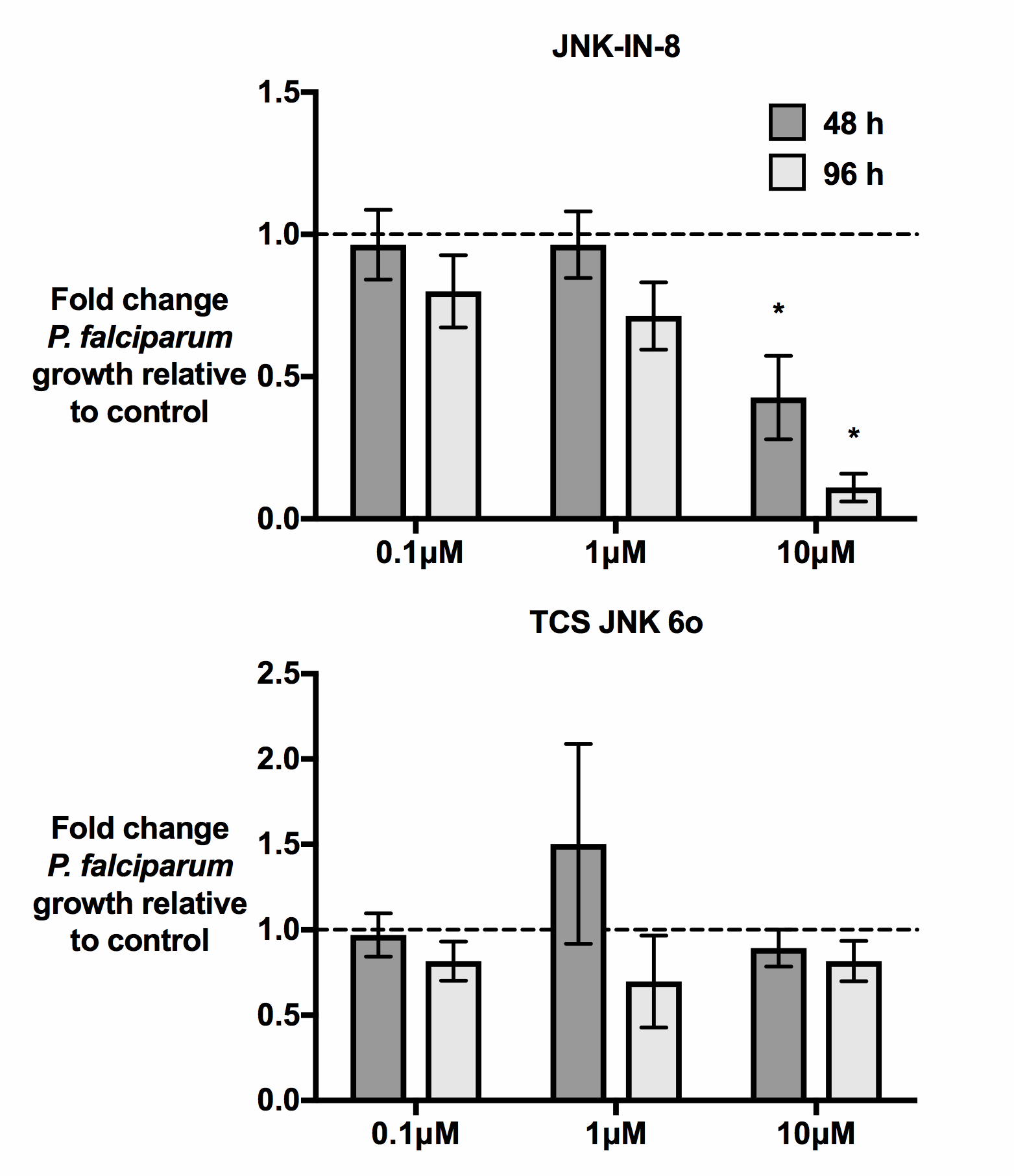

Supplement: S5 Fig — Synchronized, asexual stage P. falciparum parasites were treated with increasing concentrations of JNK SMIs and growth was evaluated at 48 and 96 h post treatment. Relative growth was normalized to parasites treated with diluent control (set at 1, dashed line). Pairwise comparisons of treatments and matched controls were analyzed by Student’s t-test, *P < 0.05. These assays were replicated four times with separate parasite culture passages. (TIFF) [file ppat.1007418.s005.tiff]

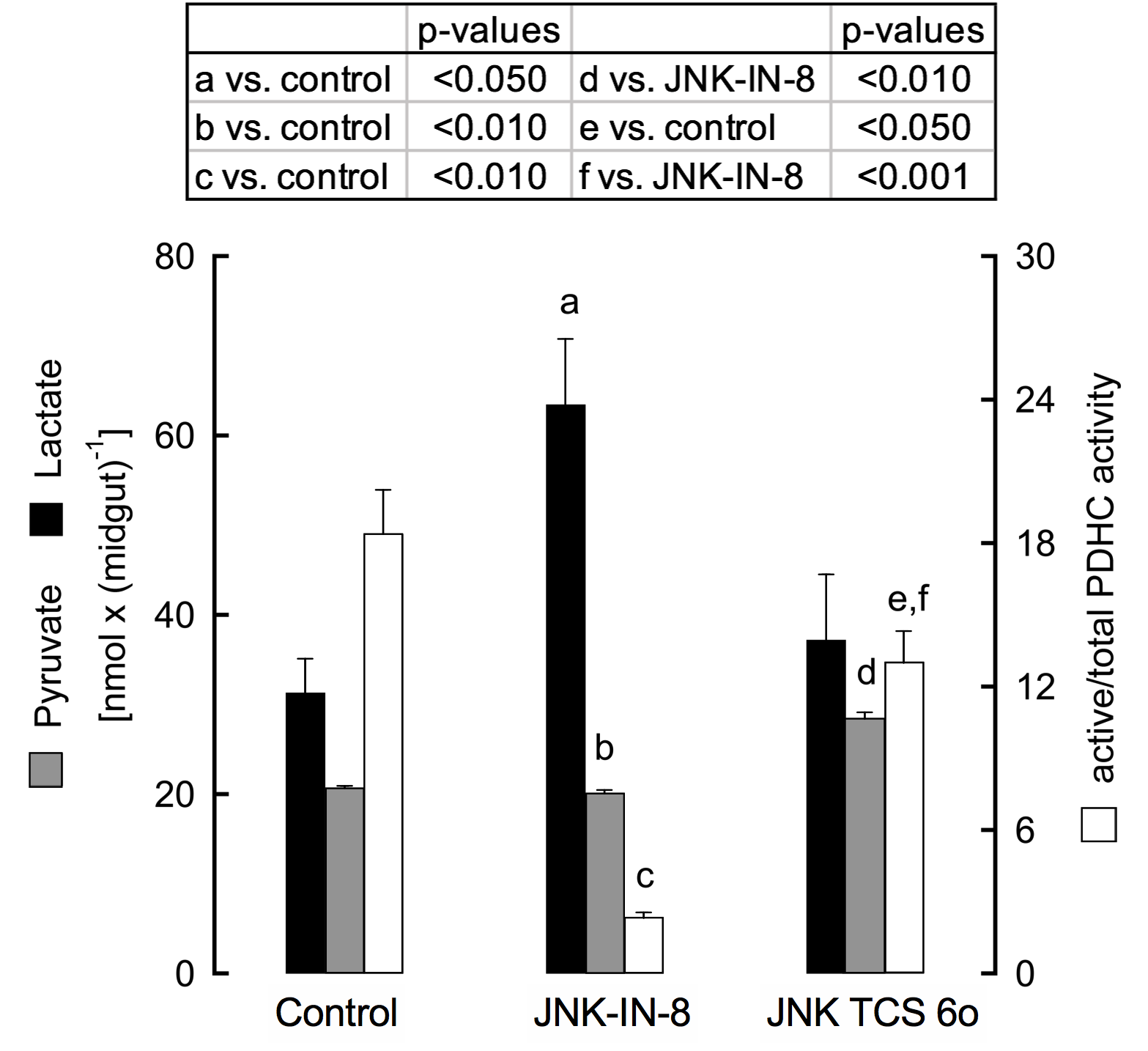

Supplement: S6 Fig — Samples were evaluated as described utilizing enzymatic assays [73]. Data were analyzed by ANOVA followed by Bonferroni’s post-hoc analysis. (TIFF) [file ppat.1007418.s006.tiff]
